# Use of an Integrated Approach Involving AlphaFold Predictions for the Evolutionary Taxonomy of *Duplodnaviria* Viruses

**DOI:** 10.3390/biom13010110

**Published:** 2023-01-05

**Authors:** Peter Evseev, Daria Gutnik, Mikhail Shneider, Konstantin Miroshnikov

**Affiliations:** 1 Shemyakin-Ovchinnikov Institute of Bioorganic Chemistry, Russian Academy of Sciences, Miklukho-Maklaya Str., 117997 Moscow, Russia; 2 Limnological Institute, Siberian Branch of the Russian Academy of Sciences, 664033 Irkutsk, Russia

**Keywords:** viral taxonomy, evolution of viruses, AlphaFold, major capsid protein, terminase, protein structure comparison

## Abstract

The evaluation of the evolutionary relationships is exceptionally important for the taxonomy of viruses, which is a rapidly expanding area of research. The classification of viral groups belonging to the realm *Duplodnaviria*, which include tailed bacteriophages, head-tailed archaeal viruses and herpesviruses, has undergone many changes in recent years and continues to improve. One of the challenging tasks of *Duplodnaviria* taxonomy is the classification of high-ranked taxa, including families and orders. At the moment, only 17 of 50 families have been assigned to orders. The evaluation of the evolutionary relationships between viruses is complicated by the high level of divergence of viral proteins. However, the development of structure prediction algorithms, including the award-winning AlphaFold, encourages the use of the results of structural predictions to clarify the evolutionary history of viral proteins. In this study, the evolutionary relationships of two conserved viral proteins, the major capsid protein and terminase, representing different viruses, including all classified *Duplodnaviria* families, have been analysed using AlphaFold modelling. This analysis has been undertaken using structural comparisons and different phylogenetic methods. The results of the analyses mainly indicated the high quality of AlphaFold modelling and the possibility of using the AlphaFold predictions, together with other methods, for the reconstruction of the evolutionary relationships between distant viral groups. Based on the results of this integrated approach, assumptions have been made about refining the taxonomic classification of bacterial and archaeal *Duplodnaviria* groups, and problems relating to the taxonomic classification of *Duplodnaviria* have been discussed.

## 1. Introduction

**Figure 1:**
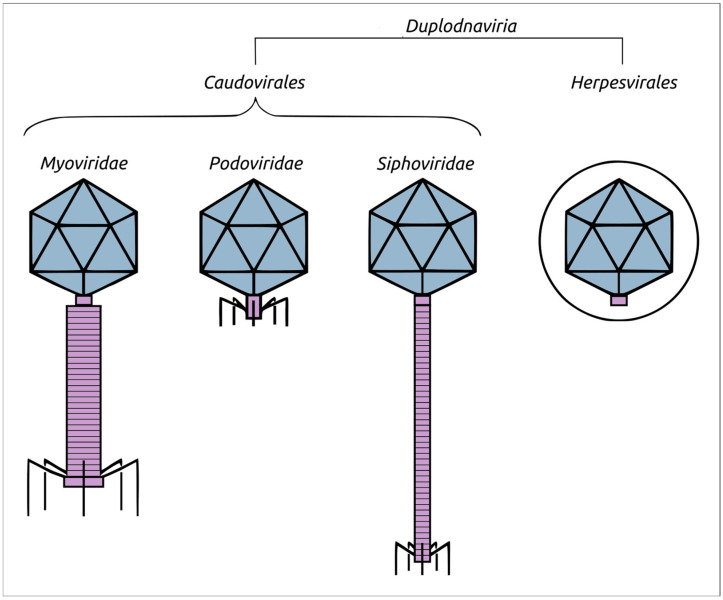
Schematic representation of the morphology of phages belonging to the former *Caudovirales* order, namely, the *Myoviridae*, *Podoviridae*, and *Siphoviridae* families, along with the evolutionarily related herpesviruses wrapped in the tegument, which is depicted as a circle. Viral capsids are coloured in slate, while tails and portal–vertex complexes are purple.

In recent years, the taxonomy of viruses has undergone significant changes. Many of these changes have been related to the reclassification of viruses infecting bacteria (bacteriophages, phages), of which tailed bacteriophages with double-stranded DNA genomes constitute the most numerous group [1]. The series of taxonomic reforms began with a shift from a classification based on bacteriophage morphology to a classification based on genomic data [2]. Since the early 2000s, the growing body of genomic data has revealed a much higher genomic diversity than was previously anticipated, primarily among tailed bacteriophages [2]. Until 2019, tailed bacteriophages were grouped within the order *Caudovirales*, which included three families, namely, *Myoviridae*, *Podoviridae* and *Siphoviridae*. Those families were created based on differences in phage morphology. Phages having a myoviral morphology (myoviruses) possess long contractile tails, while siphoviruses have flexible non-contractible tails and podoviruses possess short non-contractible (expandable) tails (Figure 1).

Traditional classification based on morphology has drastically changed, in the last several years, with the former *Caudovirales* order and the *Myoviridae*, *Podoviridae* and *Siphoviridae* families being abolished. In 2019, a decision of the Executive Committee of the International Committee on Taxonomy of Viruses (ICTV) increased the number of ranks of the taxonomic classification of viruses to fifteen [3]. Currently, the classification approved by ICTV places tailed phages, head-tailed archaeal viruses and evolutionarily related herpesviruses [4] in realm *Duplodnaviria* and kingdom *Heunggongvirae*. Herpesviruses belong to the phylum *Peploviricota* and class *Herviviricetes*, while bacterial phages and head-tailed archaeal viruses are attributed to the phylum *Uroviricota* and class *Caudoviricetes* [5]. However, classification within the class *Caudoviricetes* of the level of lower taxonomic ranks has not yet been fully formalised and is in a state of discovery and refinement. Currently, the class *Caudoviricetes* comprises four orders, 47 families, 98 subfamilies, 1907 genera and 3301 species. Most families and other lower-ranked taxa are not assigned to orders, which contain only 14 families.

The new ranking hierarchy of virus taxonomy is based on the evolutionary relationships between viruses. This hierarchy is founded upon modern evolutionary synthesis, a development of the Darwinian approach to classification, using the achievements of genomics to identify evolutionary relations [3,6,7,8,9]. The creation of a credible picture of evolutionary relationships between viral groups is often a complex task. It is assumed that viruses are of ancient origin, and they may be the most ancient creatures on Earth [10]. The long path of evolution, following the divergence of viral groups, led to many mutations in genes, which makes it difficult to identify reliable phylogenetic relationships. The problem can be partially solved by using conserved genes [11], concatenated sequences of conserved genes [12] or an analysis of protein folding and structural similarity [13]. Another problem with building the classification scheme of bacteriophages is the phenomenon of genetic mosaicism accompanying the evolution of bacteriophages, especially temperate bacteriophages, due to the modular nature of phage evolution [14,15,16,17,18]. It is difficult to solve the latter problem by merely tracing the evolutionary history of individual proteins and genes; nevertheless, this history is very important for understanding the mechanisms of viral evolution.

The structural resemblance between proteins is widely used to estimate evolutionary relationships between proteins that show little or no homology in their amino acid sequences [13,19,20]. The structural similarity between two proteins can be assessed by using the root-mean-square-deviation (RMSD) in their best-superimposed atomic coordinates, or by using other, more advanced, metrics, such as the template modelling score (TM-score) or the DALI Z-score [21,22,23]. Previously, the clusterisation of experimentally determined structures of major capsid proteins applying the DALI Z-score has been used to illustrate the common origin of several main viral groups and to cluster prokaryotic viruses [13,24]. However, for most *Caudoviricetes* families, such clusterisation is not possible, since the structures of conserved proteins for most viruses are not determined. Recent advances in protein structure prediction methods, which in some cases can give results close to those derived experimentally, encourage the use of new deep learning techniques to study the evolutionary history of protein structures. Previously, a similar approach was used to deduce patterns of the evolution of phage tail sheath proteins, but this was not tested for other viral conserved proteins, including the major capsid protein and terminase, which serve as markers of evolutionary relations between phages and are often used for taxonomic purposes [25].

In this study, the structures of the major capsid proteins (MCPs) and the ATPase subunits of terminase, encoded in the genomes of representatives of ICTV’s approved families of kingdom *Heunggongvirae*, were predicted using the winner of the CASP14 Award, AlphaFold [26], and further these predictions have been used for comparative analysis. This analysis was combined with different phylogenetic examinations, and suggestions were made about possible taxonomic updates. In addition, major capsid protein structures were modelled with another deep learning algorithm, RoseTTAFold, and the quality of predictions was assessed.

## 2. Materials and Methods

### 2.1. Data Collection and Annotation of Sequences

Viral genomes and protein sequences were downloaded from the NCBI GenBank [27] and UniProt [28] databases. Protein structures were downloaded from the Research Collaboratory for Structural Bioinformatics Protein Data Bank (RCSB PDB) [29]. Viral genomes were re-annotated with the assistance of Glimmer 3.0.2 [30], which was used for open-reading frames (ORFs) detection. Protein functions were predicted using a BLAST homology search [27] and HHM-HHM-motif comparison using the HHpred server [31].

### 2.2. Protein Modelling and Quality Assessment

All protein structures were modelled with AlphaFold 2.2.4 (AF) [26], using full databases and the command line parameter *--monomer_casp14*, matching the CASP14 configuration. Spatial structures of 53 *Caudoviricetes* major capsid proteins (MCPs) and encapsulin were also modelled with RoseTTAFold [32], with default settings using the Robetta server [33]. The best-ranked structures were selected for further study. Quality assessment of protein structures was performed using deep learning framework DeepAccNet [34], applying the default settings. Protein structures were superimposed and visualised using Pymol 2.5.4 (Schrödinger Inc., NY, USA) [35].

### 2.3. Structural Alignment and Scoring the Structural Similarity

Structure comparison was performed using the DALI server [36] and the mTM-align package [37], with default settings. Structural similarity was evaluated with the DALI Z-score [21] and the TM-score [22]. A structural similarity matrix was obtained using the DALI server. Phylogenetic trees based on structural similarity were obtained with the built-in DALI tools and the PHYLIP Phylogeny Inference Package 3.6 [38], using the neighbour-joining clustering method. Multiple sequence alignments based on structural similarity were obtained with mTM-align.

### 2.4. Phylogenetic Analysis Employing Primary Sequences of Proteins

Multiple alignments of primary amino acid sequences were obtained with Clustal Omega 1.2.3 [39], with (ten refinement iterations, evaluating full distance matrix for initial and guide trees) settings, MAFFT 7.48 [40] with default settings and using the L-INS-i algorithm, and MUSCLE 3.8.425 [41] with default settings. The phylogenetic trees based on the alignments of proteins’ primary sequences were constructed using RAxML-NG 1.1.0 [42] and the raxmlGUI 2.0.10 graphic interface [43] with (--tree rand{10} --bs-trees 1000) settings and applying the best protein model found with ModelTest-NG 0.1.7 [44]. The robustness of the RAxML-NG 1.1.0 trees was assessed using bootstrapping and calculations of transfer bootstrap estimation (TBE) support [45].

### 2.5. Comparative Analysis of Phylogenetic Trees

Pairwise comparison of best-scoring phylogenetic trees constructed with RAxML-NG 1.1.0, based on Clustal Omega 1.2.3, mTM-align, MAFFT 7.48 and MUSCLE 3.8.425 alignments, and dendrograms obtained with DALI and mTM-align structural comparisons, was performed using the ETE 3.1.2 toolkit [46] with an “unrooted tree” setting. Robinson–Foulds normalised distances (nRFs) [47] were used to compute distances between the trees and construct matrices for heatmaps. The heatmaps were visualised with a Python Plotly Express module 5.11.0.

### 2.6. VIRIDIC Intergenomic Comparison and GRAViTy Dendrogram

Comparison of intergenomic similarities was conducted with the VIRIDIC tool [48], using the default settings. The proteome-based GRAViTy dendrogram was obtained with the GRAViTy server (https://gravity.cvr.gla.ac.uk accessed on 1 November 2022) [49,50], using the database DB-B: Baltimore Group Ib-Prokaryotic and archaeal dsDNA viruses (VMRv34) and genomic sequences of representative viruses. The dendrogram was visualised with iTOL [51].

## 3. Results

### 3.1. Modelling Structures of Major Capsid Protein and ATPase Subunit of Terminase

#### 3.1.1. Selected Viral Groups

In early September 2022, the list of *Duplodnaviria* taxa approved by the ICTV encompassed 50 families. Of these, 47 families contained bacterial and archaeal head-tailed viruses and three families included eukaryotic herpesviruses. One representative of each family was picked from the list of viruses published on the ICTV website. In addition, several bacteriophages of general or particular interest, which were not assigned to the approved families, were taken to be studied. They comprised three jumbo phages, which presumably presenting ancient early diverged groups, two phages (phage λ and phage HK97) that had played an important role in viral research and two phages (*Curtobacterium* phage Ayka and *Pseudomonas* phage MD8) that were not assigned to particular families, which had been analysed in the authors’ previous research [18,52]. The jumbo phages mentioned above were the first isolated jumbo phage, *Phikzvirus phiKZ* (*Pseudomonas* phage phiKZ) [53,54], *Donellivirus gee* (*Bacillus* phage G), which is an isolated phage with the largest known genome [55], and the phage with the largest known genome predicted by metagenome analysis, LacPavin_0818_WC45 [56] (Table 1).

Use of the ICTV-recommended intergenomic similarity comparison VIRIDIC tool [48] did not show any meaningful genomic likeness between the representative viruses (Supplementary Figure S1), indicating that the genus threshold of 70% nucleotide identity [2] cannot be applied to these viruses.

#### 3.1.2. Major Capsid Protein Modelling

Experimentally determined, and bioinformatically predicted, 57 genes encoding the major capsid proteins (MCPs) have been translated and modelled by AlphaFold (AF). The best-ranking structures were taken for further analysis. For use as an outgroup for the phylogenetic analysis, the structure of *Thermotoga maritima* encapsulin (PDB code 7K5W), which has a high structural resemblance to *Heunggongvirae* MCPs [57], was also modelled with AlphaFold. In addition, 53 MCPs belonging to viruses assigned to phylum *Uroviricota* and class *Caudoviricetes* (phages and head-tailed archaeal viruses) were modelled with RoseTTAFold to evaluate the prediction quality of the programs.

Interestingly, an examination of GenBank annotations indicated the absence of MCP annotations in several cases. Furthermore, examination of annotations using HHpred indicated errors in annotations for selected representatives of *Duneviridae* and *Helgolandviridae* families. The HHpred examination of MCP sequences obtained by the translation of corresponding predicted genes of *Hacavirus HCTV1* (*Haloarcula californiae* tailed virus 1, order *Thumleimavirales*, family *Soleiviridae*) found no meaningful similarities with viral capsid proteins, which was also the case with *Phikzvirus phiKZ*, but subsequent modelling of suggested MCP of *Hacavirus HCTV1* and experimental data for *Phikzvirus phiKZ* confirmed the functions of these proteins.

**Figure 2:**
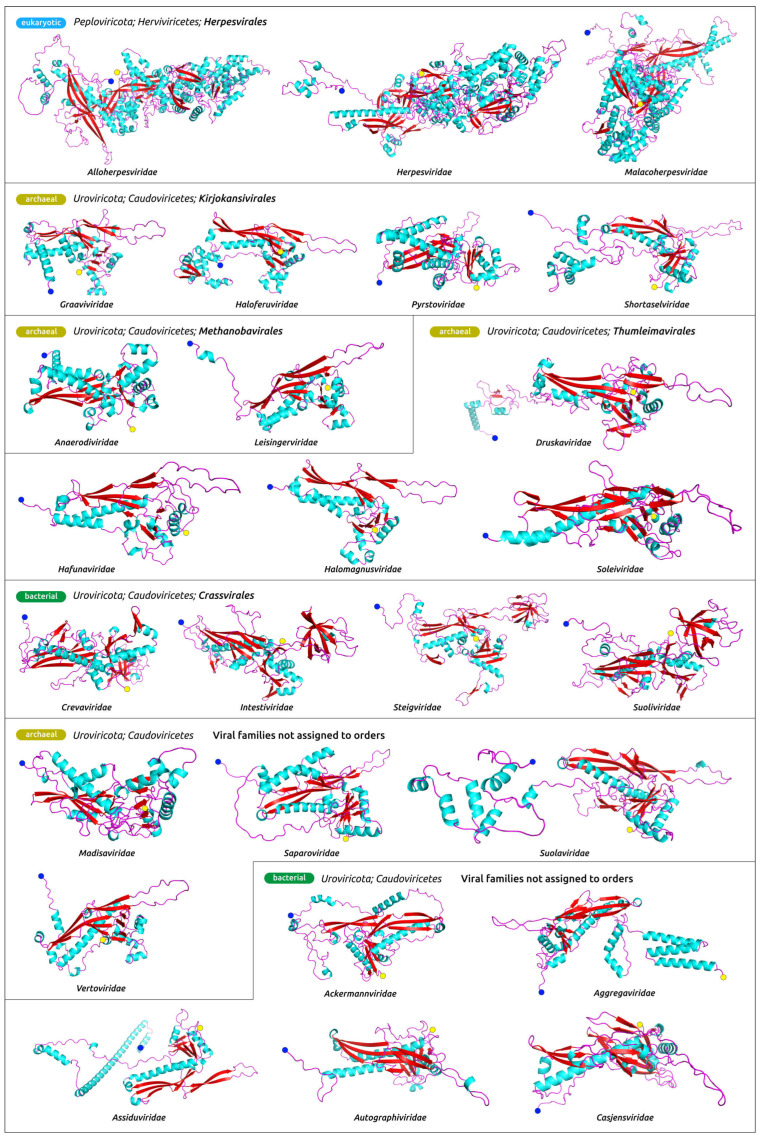
Structural models of the product of the whole genes encoding for the major capsid proteins in the genomes of representative viruses (Table 1), obtained with AlphaFold. The N-terminus is labelled with a blue circle and the C-terminus is labelled with a yellow circle. Other predicted MCP structures are shown in Supplementary Figure S2.

**Figure 3:**
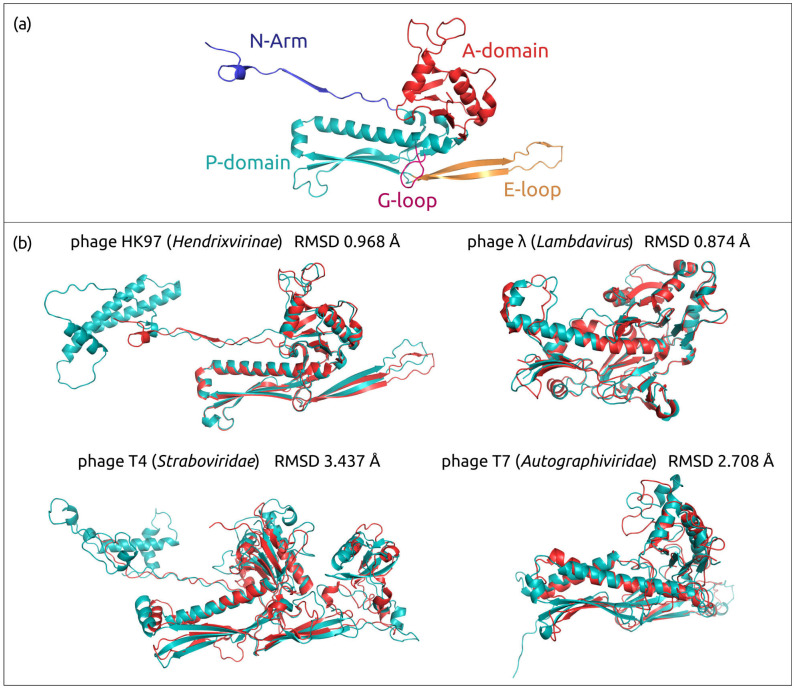
(**a**) The HK97 fold and its common features coloured as indicated in the figure (according to Duda et al. [67]). The diagram is of the mature HK97 capsid (PDB code 1OHG_A). (**b**) Superimposition of AF models (depicted in teal) and RCSB PDB structures (depicted red) belonging to the same viruses: 1OHG (phage HK97, the mature capsid), 7SJ5 (λ, major capsid protein mutant in the pre-assembly conformation), 5VF3 (T4, mutant MCP in the isometric capsid), 3J7V (T7) and corresponding RMSD values.

All the models (Figure 2 and Supplementary Figure S2) contain characteristic HK97-fold (Figure 3a), named after *Byrnievirus HK97* (*Escherichia* phage HK97), including its conserved elements, the A-domain (axial domain), the P-domain (peripheral domain), the E-loop (extended loop) and the N-arm (N-terminal arm). Most models contain additional elements found in different HK97-like capsid proteins, such as the G-loop. The modelled proteins often contain other domains, or subdomains, that can also be explained by the presence of protease and scaffolding protein domains in the translated sequences. Protease and scaffolding protein are essential for capsid assembly and can be encoded in a single gene, but are absent in mature capsids [58,59,60,61]. The superimposition of modelled AF structures of phages HK97, λ, T4 and T7, and experimentally determined corresponding structures 1OHG (HK97, the mature capsid) [62], 7SJ5 (λ, major capsid protein mutant in the pre-assembly conformation) [63], 5VF3 (T4, mutant MCP in the isometric capsid) [64], 7VS5 (T4, MCP in the expanded head structure) [65] and 3J7V (T7, MCP in the DNA-free procapsid state) [66], showed RMSD values of 0.968 Å, 0.874 Å, 3.437 Å, 0.763 Å and 2.708 Å, respectively (Figure 3b). These values are lower than the corresponding experimental resolution values (3.45 Å, 2.69 Å, 3.45 Å, 3.40 Å and 4.60 Å, respectively).

The most complex structural architecture was found in MCP models of herpesviruses and Jumbo phage phiKZ. Interestingly, *Pseudomonas* phage MD8 also featured a comparatively complicated architecture. As was suggested earlier, for this phage, a single gene encodes for major capsid protein, protease and scaffolding proteins as a single propeptide [18].

#### 3.1.3. ATPase Subunit of Terminase Modelling

The ATPase subunits of terminase (terminase, large subunit of terminase, TerL) were modelled in a similar way. The terminase genes were extracted from the annotations of representative genomes and translated. The terminase (gene IVa2) of *Human adenovirus C*, exploiting a mechanism of genome packaging similar to herpesviruses and tailed phages [68], was also modelled with AF in order to use it as an outgroup in phylogenetic analyses.

**Figure 4:**
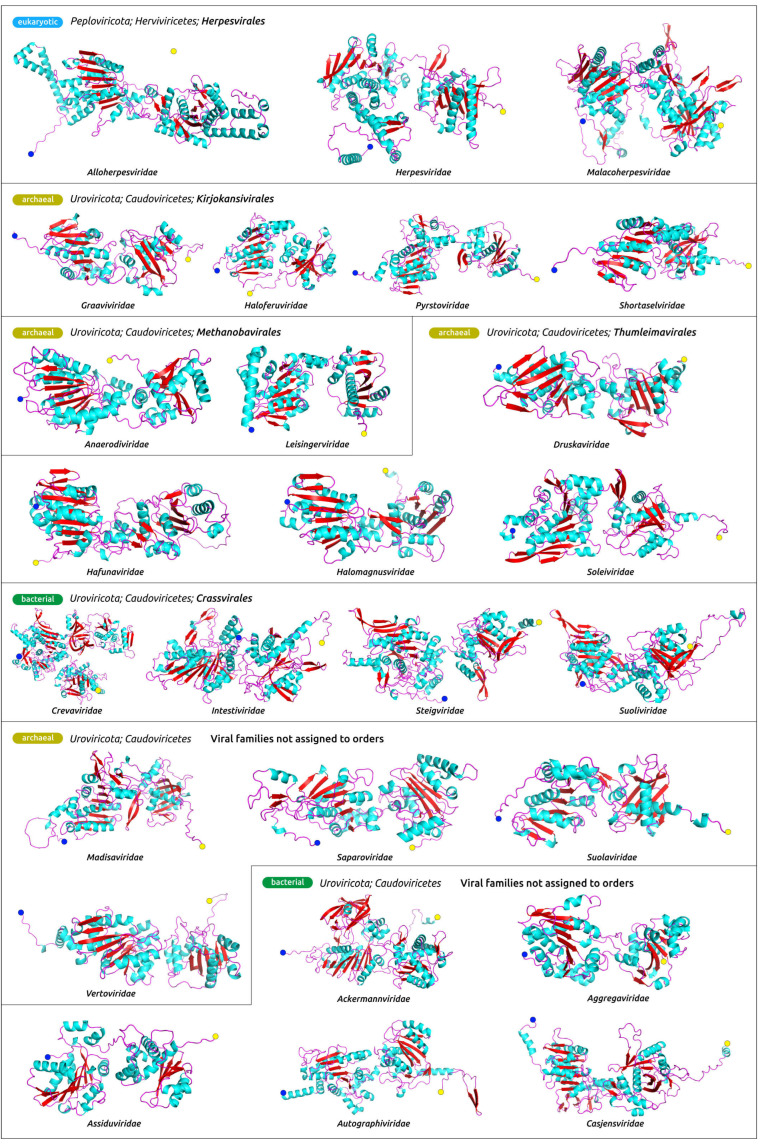
Structural models of the product of the whole genes encoding for the ATPase subunit of terminase in the genomes of representative viruses (Table 1), obtained with AlphaFold. The N-terminus is labelled with a blue circle and the C-terminus is labelled with a yellow circle. Other predicted terminase structures are shown in Supplementary Figure S3.

**Figure 5:**
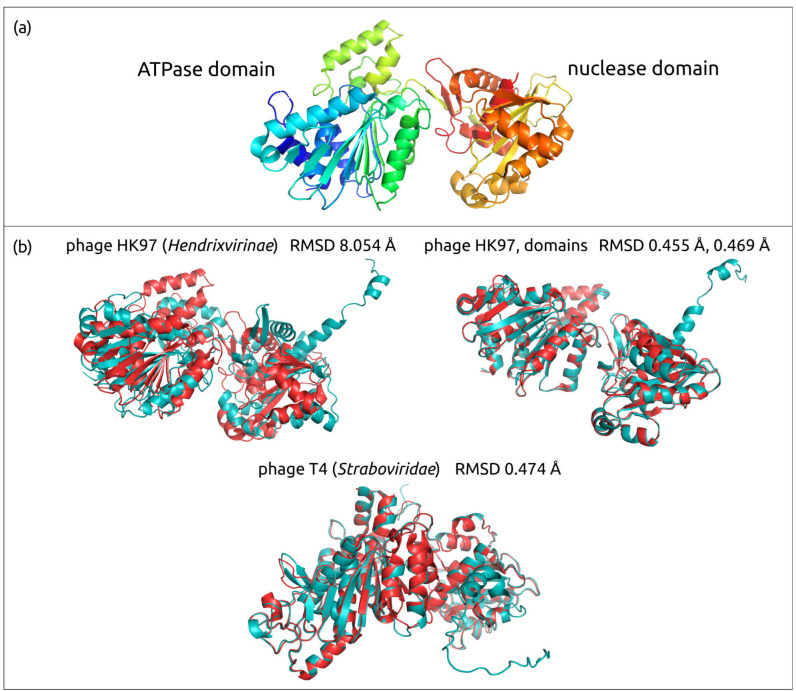
(**a**) Ribbon diagram of the phage HK97 large subunit of terminase (PDB code 6Z6D), coloured based on a rainbow gradient scheme, where the N-terminus of the polypeptide chain is coloured blue and the C-terminus is coloured red. (**b**) Superimposition of AF models (depicted in teal) and RCSB PDB structures (depicted in red), belonging to the same viruses: 6Z6D (phage HK97, using the whole model and separated ATPase and nuclease domains), 3CPE (phage T4, using the whole model) and corresponding RMSD values.

The structural architecture of *Heunggongvirae* TerL reflects the function of this protein. Typical ATPase subunits of terminase include the N-terminal adenosine triphosphatase (ATPase) domain that drives DNA translocation and the C-terminal endonuclease domain that cleaves the concatemeric genome at both initiation and completion of genome packaging [69]. The ATPase domain (ATDP) contains a five-stranded, parallel β-sheet in the canonical ASCE fold sandwiched between several α-helices, which is easily recognisable in the models (Figure 4, Figure 5 and Figure S3), and additional β-strands that are unique in viral terminases [70].

The superimposition of the TerL model of phage HK97 and experimentally determined structure 6Z6D produced the RMSD value of 8.054 Å (the experimental accuracy was 2.20 Å), and the superimposition of the phage T4 TerL model and experimentally determined structure 3CPE produced the RMSD value of 0.474 Å (the experimental accuracy was 2.80 Å). An inspection of the HK97 model and the X-ray determined structure indicated that the comparatively high RMSD was due to the predicted orientation of ATPase and nuclease domains relative to each other. A superimposition using the separated domains without the linker part yielded the RMSD values of 0.455 Å for the ATPase domain and 0.469 Å for the nuclease domain.

#### 3.1.4. Evaluation of Models’ Accuracy

**Figure 6:**
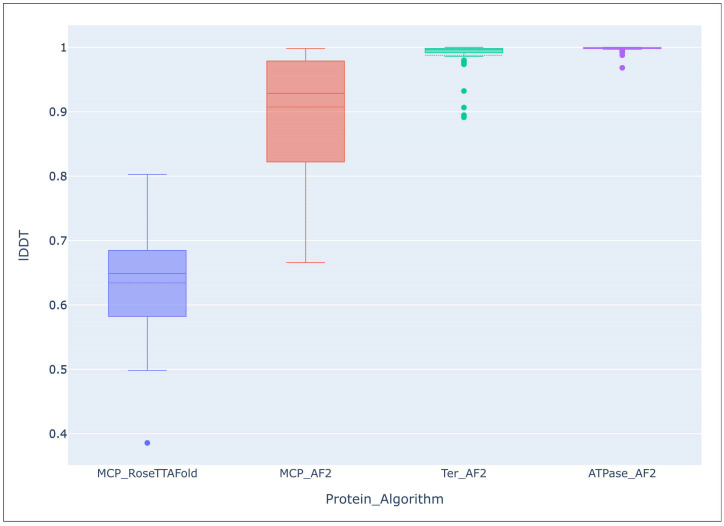
Comparison of the overall accuracy of predictions made with the Local Distance Difference Test (lDDT), using the DeepAccNet accuracy predictor. MCP_RoseTTAFlold–RoseTTAFlold models of the MCP, MCP_AF2–AlphaFold models of the MCP, Ter_AF2–terminase ATPase subunits’ models predicted with AlphaFold, ATPase_AF2–ATPase domain of terminase ATPase subunits’ models predicted with AlphaFold.

Overall accuracy predictions were assessed with the Local Distance Difference Test (LDDT), using the DeepAccNet accuracy predictor. Comparison of the average lDDT score of the 54 AF models of MCP and TerL indicated a mostly high level of accuracy of predictions, and that structure prediction of TerL was more accurate than that of MCP, (lDDT TerL mean: 0.988, median: 0.996, q1: 0.991, q3: 0.999; lDDT MCP mean: 0.907, median: 0.929, q1: 0.822, q3: 0.970). The average lDDT of the ATPase domains extracted from the TerL models was even higher, (mean: 0.998, median: 0.999, q1: 0.998, q3: 0.9997). The evaluation of RoseTTAFold models of the same 53 MCPs showed a lower accuracy of prediction, (lDDT mean: 0.634, median: 0.649, q1: 0.582, q3: 0.685), than with the AlphaFold models (Figure 6).

### 3.2. Comparisons of Models’ Structural Similarity

#### 3.2.1. Structural Comparisons Using DALI

**Figure 7:**
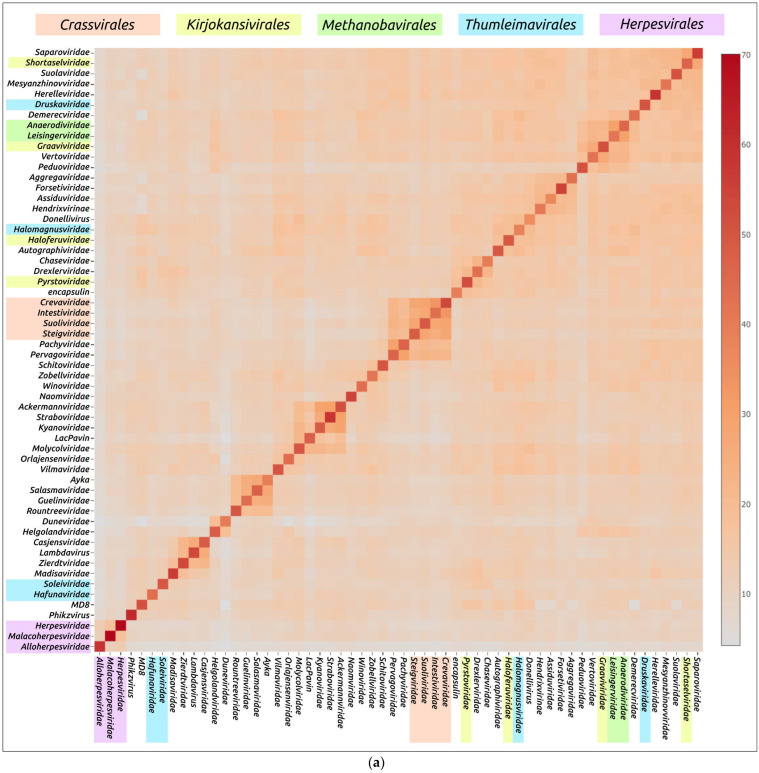

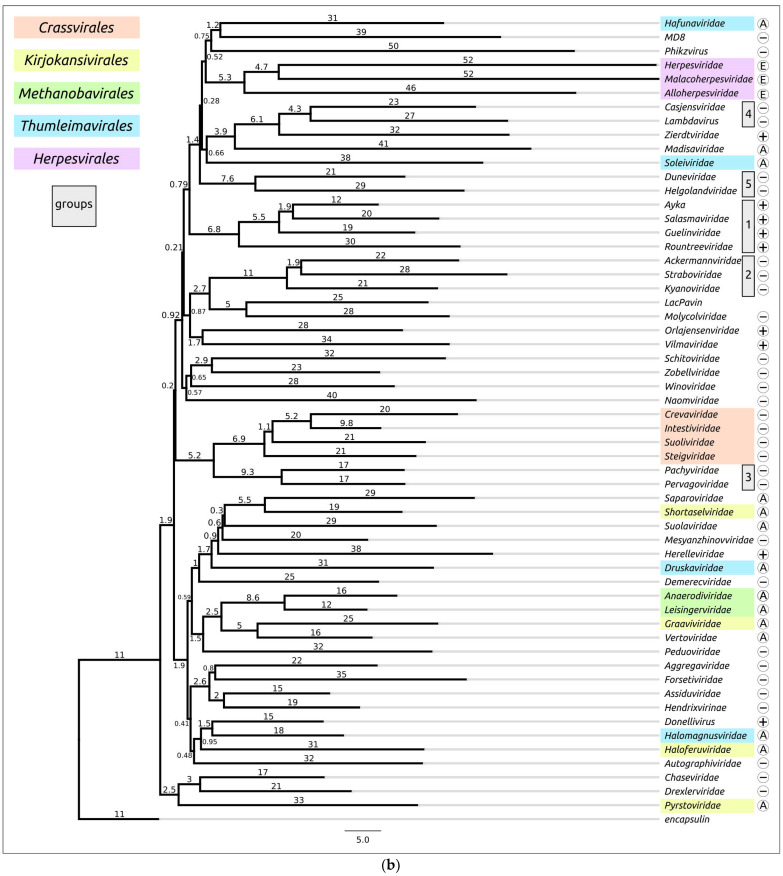
Matrix (**a**) and dendrogram (**b**) based on the pairwise Z-score comparisons of 57 major capsid proteins and encapsulin AF models, using DALI. The branch lengths are measured using the DALI Z-score and the tree was rooted to encapsulin. “A”—archaeal viruses, “E”—eukaryotic viruses, “+”—phages infecting Gram-positive bacteria, and “-”—phages infecting Gram-negative bacteria.

An evaluation of the structural similarity of proteins including viral major capsid proteins, using DALI, was carried out to investigate the evolutionary relationships and classification of protein fold [13,36]. A DALI analysis using the AF models of major capsid proteins (Figure 7) demonstrated clustering of all four bacteriophages of the *Crassvirales* order (families *Crevaviridae*, *Intestiviridae*, *Steigviridae* and *Suoliviridae*) and both head-tailed archaeal viruses of the *Methanobavirales* order (*Anaerodiviridae* and *Leisingerviridae* families). At the same time, the MCPs of the head-tailed archaeal viruses of the *Kirjokansivirales* and *Thumleimavirales* orders did not form distinct clusters. The MCP models of representatives of the *Herpesvirales* order (*Alloherpesviridae*, *Herpesviridae* and *Malacoherpesviridae* families) were grouped together, but did not show such high similarities as MCPs of the *Crassvirales* and *Methanobavirales* orders. The results of DALI clustering using the structures with removed parts, approximately corresponding to the protease and scaffolding domains, were similar to those of full-sized models (Supplementary Figure S4).

In addition, DALI indicated noticeable structural resemblances of representative MCPs for other families, including:

- The bacteriophages of *Guelinviridae*, *Rountreeviridae* and *Salasmaviridae* families, and a novel *Curtobacterium* phage Ayka; (from now on, in this study, these will be referred to as group 1);
- The bacteriophages of *Ackermannviridae*, *Kyanoviridae* and *Straboviridae* families (group 2);
- The bacteriophages of *Pachyviridae* and *Pervagoviridae* families (group 3), making a subcluster of a larger cluster that includes the *Crassvirales* phages;
- The bacteriophages of the *Casjensviridae* family and *Lambdavirus lambda* (group 4);
- The bacteriophages of *Duneviridae* and *Helgolandviridae* families (group 5).

Some of these observations can be biologically meaningful, reflecting the common origin and lifestyle of viruses. Group 1 comprises the so-called “ϕ29-like” lytic phages with a podoviral morphology and similar genome size of about 16–20 kb, infecting Gram-positive bacteria [52,71,72,73]. The *Guelinviridae*, *Rountreeviridae* and *Salasmaviridae* families were proposed in 2020, clarifying the taxonomic classification of ϕ29-like viruses [5]. Group 2 includes phages with large genomes of about 150–200 kb, which were described earlier as “T4-like” phages [74,75,76]. MCP models of unclassified Jumbo-phage LacPavin (genome size 735 kb) and *Maribacter* phage Colly_1 (*Molycolviridae* order, genome size 735 kb) also showed some resemblance to group 2 models.

Viruses of group 3 [77,78] infect flavobacteria (phylum *Bacteroidota*) and have genomes of a similar size (about 73 kb) and GC-content (Table 1). A BLAST search using the GenBank PHG database indicated the relatedness of the MCPs of *Pachyviridae* and *Pervagoviridae* representative phages (Bit-score of more 130), but did not reveal homologies with the *Crassvirales* phages, which infect human gut symbiont *Bacteroides* [79,80]. Both group 3 and *Crassvirales* phages have a podoviral morphology. Phages of group 4 have genomes of 49–67 kb and infect *Enterobacterales* (*Salmonella* phage χ of *Casjensviridae* family [81] and phage λ [82]). Group 5 includes the phages *Flavobacterium* phage 1H (*Duneviridae* family) [83] and *Polaribacter* phage Leef_1 (*Helgolandviridae* family) [78], infecting flavobacteria. The phages have genomes of similar size (about 38–39 kb) and features with a siphoviral morphology.

It is noteworthy that, according to the DALI comparisons of MCP structural similarities, the archaeal head-tailed viral families do not form one or two distinct clusters. They are often grouped with bacteriophages or do not show similarities with any other families.

**Figure 8:**
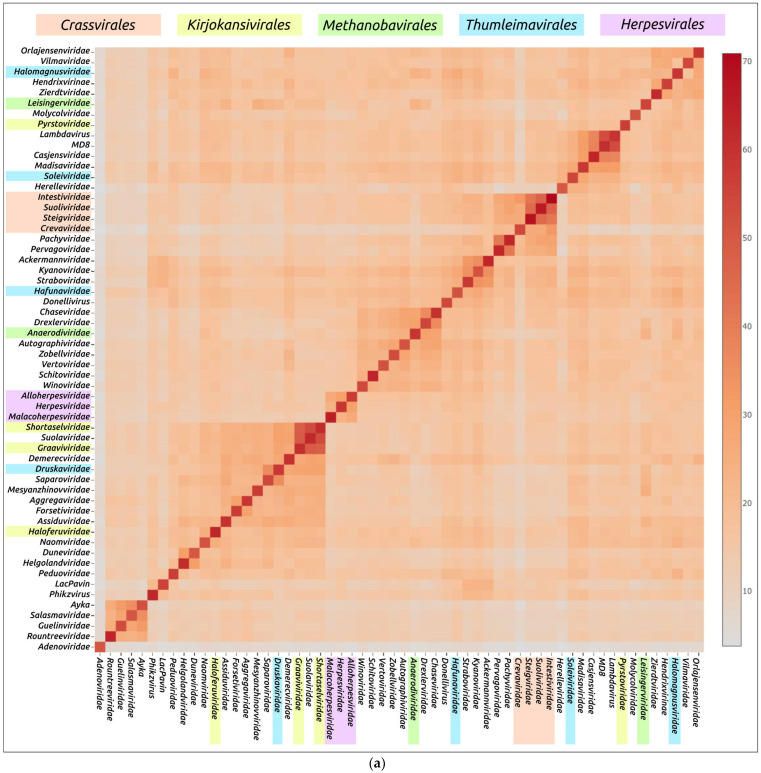

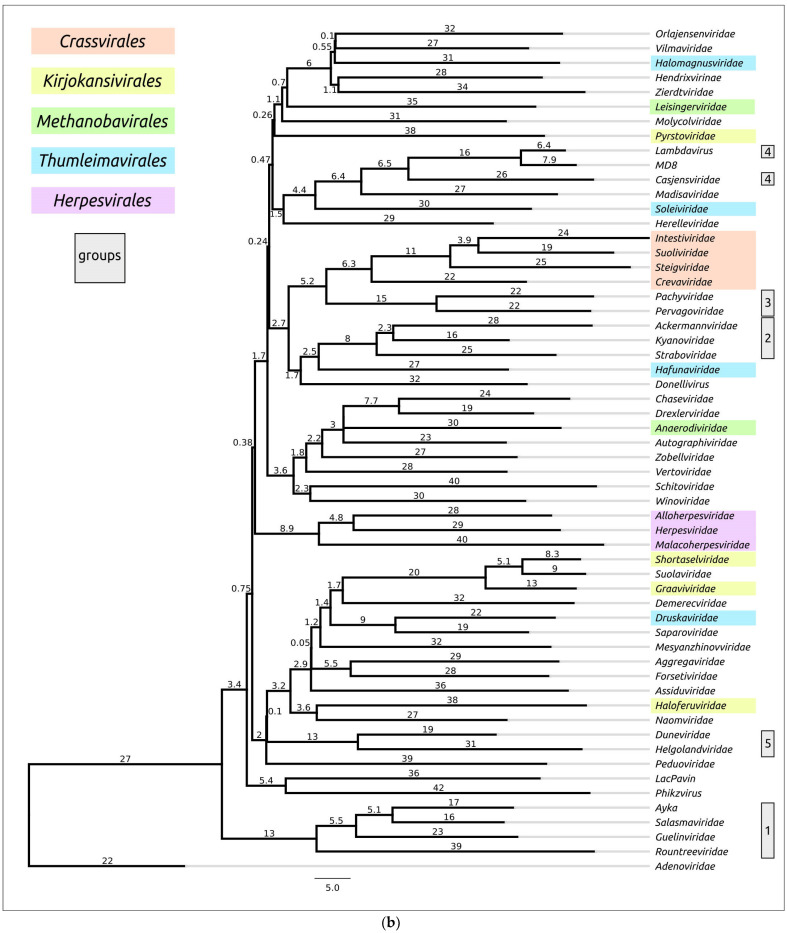
Matrix (**a**) and dendrogram (**b**) based on the pairwise Z-score comparisons of 58 AF models of ATPase subunit of terminase including an *Adenoviridae* terminase, using DALI. The branch lengths are measured using the DALI Z-score and the tree was rooted to *Adenoviridae*.

**Figure 9:**
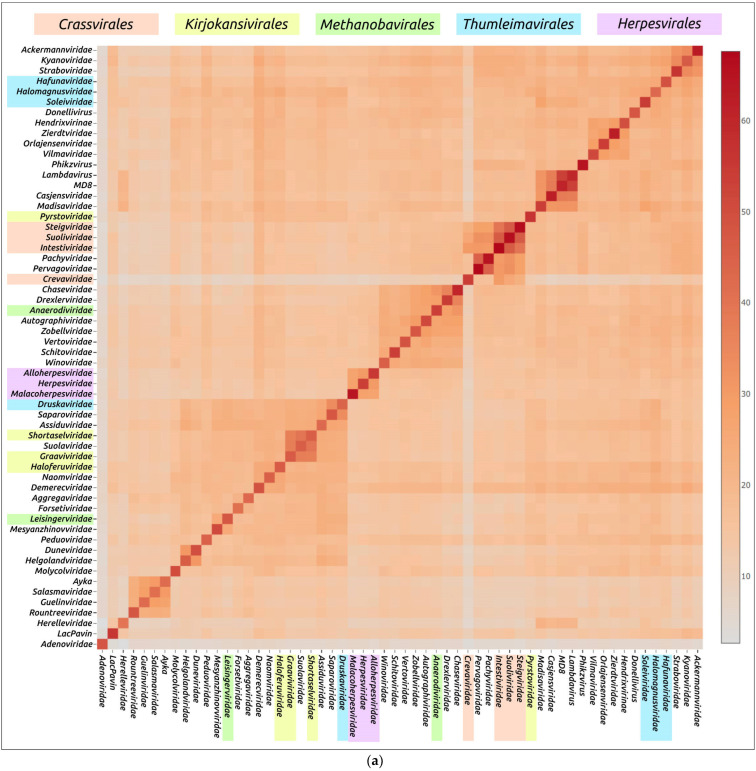

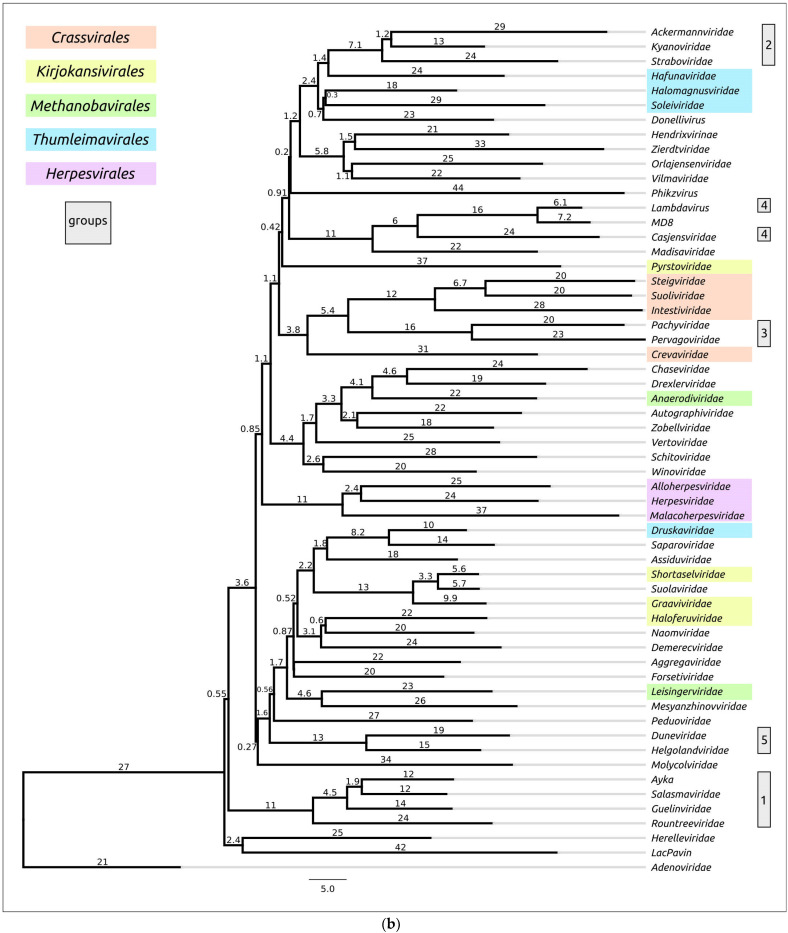
Matrix (**a**) and dendrogram (**b**) based on the pairwise Z-score comparisons with DALI, using 57 ATPase domain structures extracted from TerL AF models including an *Adenoviridae* terminase. The branch lengths are measured using the DALI Z-score and the tree was rooted to *Adenoviridae*.

The DALI analysis performed using the modelled structures of a large subunit of terminase (TerL) and its ATPase domain (ATPD) demonstrated similar results (Figure 8 and Figure 9). The TerL (or ATPD) analysis showed differences in the DALI MCP comparison. Generally, structural similarities of representative models TerL and ATPD were greater than for MCPs, indicating the greater conservation of terminase. TerL and ATPD structural comparisons with DALI also gathered the viruses of groups 1, 2 and 3 mentioned above into distinct clusters and indicated the likeness of group 3 *Pachyviridae* and *Pervagoviridae* terminase models to *Crassvirales* terminases. Interestingly, the TerL and ATPD models of phage λ (group 4), unclassified *Pseudomonas* phage MD8, distantly related to lambdoid phages, a *Casjensviridae* family phage *Salmonella* phage χ (group 4) and archaeal head-tailed *Haloarcula hispanicatailed* virus 1 (*Madisaviridae* family) showed a distinct similarity. These viruses have a siphovirus morphology [24] and a genome size of 48–59 kb. As well as the DALI MCP comparisons, the terminase analysis indicated a complex pattern of relationships between archaeal viruses, not matching the ICTV classification.

#### 3.2.2. Structural Comparisons with mTM-Align

**Figure A1:**
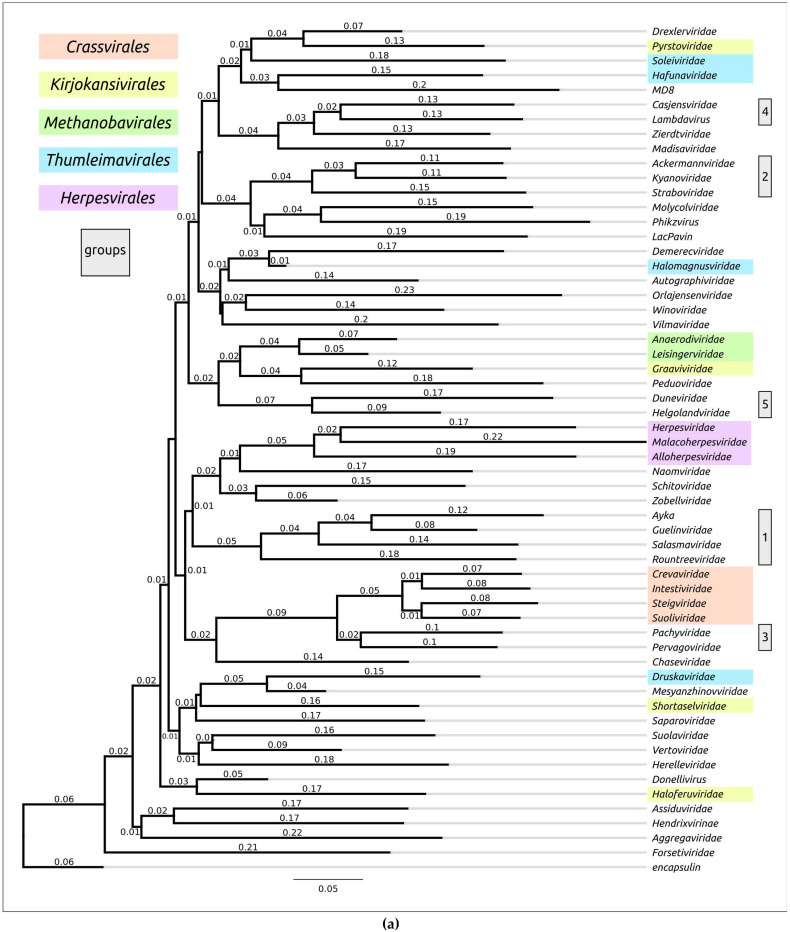

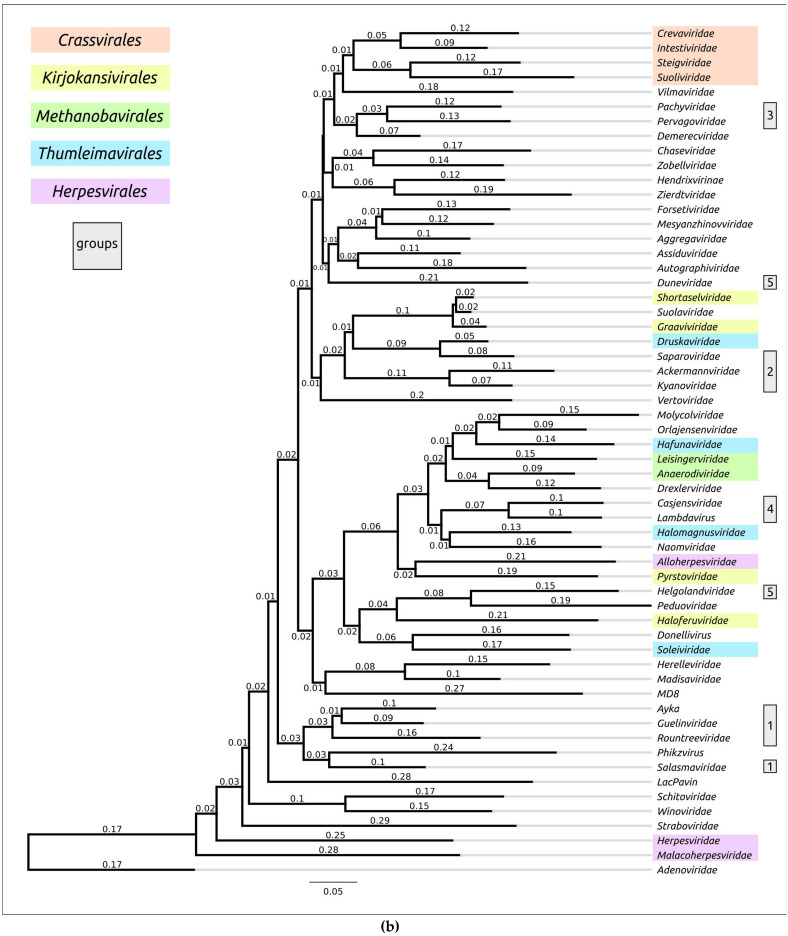

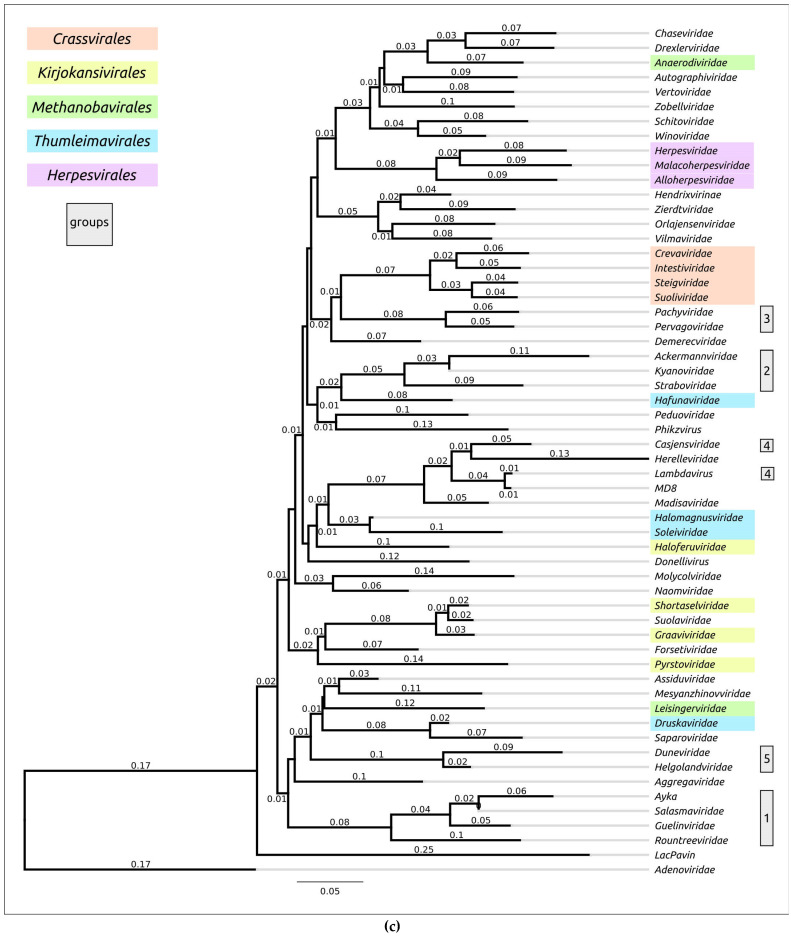
Dendrograms based on protein structure comparisons using mTM-align: (**a**) 57 major capsid protein and an encapsulin AF models. The tree was rooted to encapsulin; (**b**) 58 ATPase subunits of *Heunggongvirae* terminases and an *Adenoviridae* terminase. The tree was rooted to *Adenoviridae*; (**c**) 58 ATPase domains, extracted from AF models of ATPase subunits of *Heunggongvirae* terminases and an *Adenoviridae* terminase, and the tree was rooted to *Adenoviridae*. The branch lengths are measured with TM-scores.

The results of the mTM-align structural comparisons (Figure A1) did not match the conclusions of the DALI examinations. However, there were many similar observations concerning the similarities of the modelled structures. For example, a relatedness was shown between the MCPs and terminases of group 1, group 2 and group 3 viruses, mentioned in Section 3.2.1, and the analysis also showed the complex grouping pattern of archaeal viruses. As well as the MCP DALI tree, the mTM-align tree placed the *Methanobavirales* order families in a monophyletic branch, showing the similarity of *Lambdavirus* and *Casjensviridae* MCP models and *Hafunaviridae* and MD8 models.

The mTM-align tree constructed with the whole models of the ATPase subunit of terminase did not place all three families of the *Herpesvirales* order in a single clade, but the MCP and ATPD trees set these families in clades. The latter trees placed the *Pachyviridae* and *Pervagoviridae* structures to the clade, containing the *Crassvirales* representatives, like the DALI trees do.

### 3.3. Phylogenetic Analysis Using Amino Acid Sequences of MCP and TerL

#### 3.3.1. Phylogenetic Analysis of MCP

**Figure 10:**
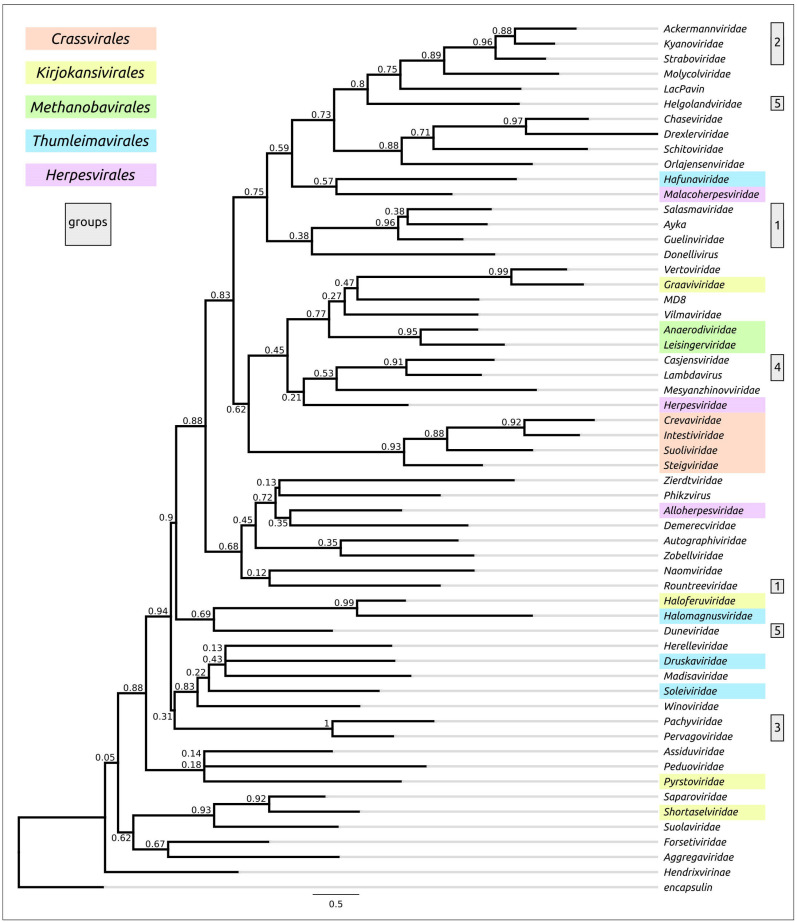
Best-scoring ML phylogenetic tree constructed with 57 amino acid sequences of major capsid protein and an encapsulin aligned with Clustal Omega. The scale bar shows 0.5 estimated substitutions per site and the trees were rooted to encapsulin.

In addition to the evidence of trees that made comparisons based on structural similarity, a comparative analysis was conducted with phylogenetic trees, which were based on alignments obtained with different algorithms (Clustal Omega, MAFFT, MUSCLE, and mTM-align) using MCP amino acid sequences (Figure 10 and Figures S5–S7); the latter analyses showed unmatching topologies. It did, however, demonstrate the common composition of some branches. Only the tree constructed using the alignment based on the structural similarity obtained with mTM-align placed all the *Herpesvirales* representatives in distinct monophyletic branches. Except for this tree, none of the trees arranged the *Crassvirales* families and the representatives of group 3 (*Pachyviridae* and *Pervagoviridae* families) in monophyletic or adjacent branches. Except for the MUSCLE tree, none of the trees placed the representatives of group 1 (ϕ29-like *Guelinviridae*, *Rountreeviridae* and *Salasmaviridae* families, and *Curtobacterium* phage Ayka) in a monophyletic branch. However, for the sequences belonging to group 2 (T4-like *Ackermannviridae*, *Kyanoviridae* and *Straboviridae* families), and those belonging to the *Methanobavirales* order’s families, the phylogenetic analyses based on amino acid sequence alignments showed results resembling those produced from structural comparisons. Apparently, low MCP sequence conservation level (MAFFT pairwise identity 6.0%) hinders the possibilities of phylogenetic analysis.

#### 3.3.2. Phylogenetic Analysis of Terminase

**Figure 11:**
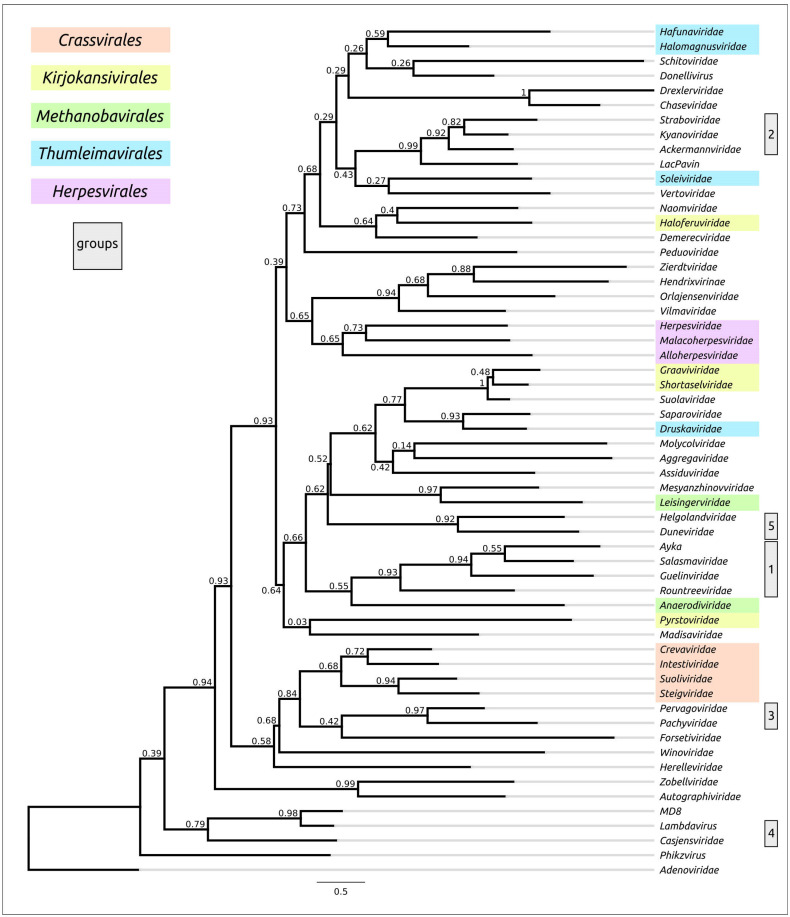
Best-scoring ML phylogenetic tree constructed with 57 amino acid sequences of ATPase subunits of *Heunggongvirae* terminases and an *Adenoviridae* terminase aligned with Clustal Omega. The numbers near the tree branches indicate the TBE support. The total number of bootstrap trees was 1000. The scale bar shows 0.5 estimated substitutions per site and the trees were rooted to *Adenoviridae*.

A phylogenetic analysis based on the amino acid sequences of the ATPase subunit of terminase TerL and the ATPase domain, identified with the assistance of AlphaFold, was conducted using alignments obtained with Clustal Omega, MAFFT, MUSCLE and mTM-align (Figure 11 and Figures S8–S10). Apparently, the level of conservatism of TerL was somewhat higher than for MCP (pairwise identity of TerL alignment by MAFFT 7.5%). Except for the MUSCLE trees, the remaining trees grouped the *Herpesvirales* and group 2 (ϕ29-like) representatives in distinct clades. Most of the trees arranged the representatives of both group 3 and order *Crassvirales* in a monophyletic branch, and all trees placed the proteins of group 1 (T4-like viruses) in a clade. However, as well as in the cases described above, the trees demonstrated different topologies. Along with all other structural and phylogenetic analyses, the terminase trees showed complex relationships between the terminases of archaeal head-tailed viruses, which did not match the current ICTV classification.

### 3.4. Analysis of Topological Congruence of Dendrograms

**Figure 12:**
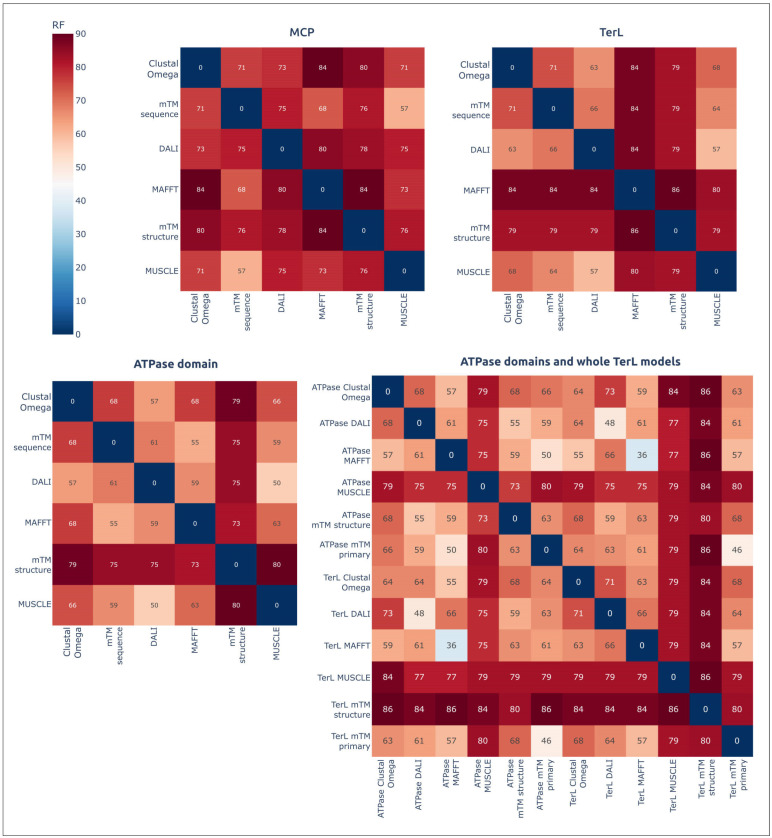
Comparisons of the topological congruence of trees, obtained using structural alignments and different amino acid sequence alignments, and also the normalised RF score shown in matrices. The designation “mTM primary” means that the tree was constructed using the alignment of amino acid sequences with mTM-align; the designation “mTM structure” means that the tree was constructed using structural similarity as measured with TM-scores.

Comparisons of the topologies of dendrograms, based on both the structural similarities and amino acid sequence alignments, using the ETE toolkit calculated high Robinson–Foulds normalised distances (nRFs), indicated the topological incongruence of the trees (Figure 12). The comparisons also indicated greater topological similarities between ATPD and TerL trees constructed using the same algorithms; topological similarities of ATPD trees were basically more pronounced. A visual comparison showed better topological similarities between the branches that had diverged comparatively recently.

### 3.5. GRAViTy Dendrogram

Evolutionary relationships between the viruses were also estimated with the GRAViTy pipeline, classifying viral groups according to the homology between viral genes and similarities in genomes’ organisation. The GRAViTy tool is recommended by the ICTV for the demarcation of high-ranked taxa [2]. The GRAViTy tree (Supplementary Figure S11) shows differences from the DALI structural similarity dendrogram and other trees, clustering two *Herpesvirales* families together with four *Thumleimavirales* families of archaeal viruses and placing both the representatives of group 3 (*Pachyviridae* and *Pervagoviridae*) and group 4 (*Casjensviridae* and *Zierdtviridae*) in distant clusters. The differences in topologies could also be due to a different composition of viruses involved in the analysis. The GRAViTy dendrogram clustered all representatives of the *Crassvirales* order, of group 1 (representatives of *Guelinviridae*, *Rountreeviridae* and *Salasmaviridae* families, and *Curtobacterium* phage Ayka), group 2 (*Ackermannviridae*, *Kyanoviridae* and *Straboviridae*) and group 5 (*Duneviridae* and *Helgolandviridae*) in corresponding groups (also containing other viruses not present in the list of 57 representative viruses) (Table 1). Nevertheless, not all representatives of the *Kirjokansivirales* and *Methanobavirales* orders were grouped according to their taxonomic classification. Interestingly, the GRAViTy dendrogram placed *Plasmaviridae* and *Helgolandviridae* representatives into one branch. *Plasmaviridae* is a family of pleomorphic enveloped viruses, not belonging to class *Caudoviricetes*, that infect *Acholeplasma* species [84].

## 4. Discussion

AlphaFold modelling of the structures of two viral conservative proteins, the major capsid protein and the ATPase subunit of terminase, has demonstrated high predictive accuracy. This accuracy exceeded that of RoseTTAFold, another deep-learning algorithm, identifying AlphaFold as being preferred to RoseTTAFold for such purposes. Using predicted accuracy alone, however, it is difficult to judge the extent to which the models correspond to real structures. It should also be pointed out that the native state of viral proteins can change, in different states of the viral particle (e.g., empty, full, expanded capsids) and at different stages of viral particle assembly [64,65,85,86]. The correlation between structural similarity and sequence identity is not absolute, due to conformational plasticity, mutations, solvent effects and ligand binding [87]. Most of these limitations refer to analyses that also employed experimentally determined structures, but they can be exacerbated by structural prediction errors. Therefore, it seems to be difficult to forecast the effectiveness of using AlphaFold for the analysis of structural similarity and evolutionary history based on the resemblance of predicted structures alone. In addition, as shown in this study, different algorithms of structural comparison can lead to different results. Unfortunately, some limitations are inherent not only in structural analysis, but also in analyses based on primary amino acid protein sequences. Recovering evolutionary history using amino acid alignments has its own problems, related to high mutation rates, the details of molecular evolution and depends on algorithms and methods [88,89,90,91], as was seen in the phylogenetic studies performed in this work. Thus, an integrated approach involving the evaluation and comparison of various methods, including structural- and sequence-based phylogenies, genome organisation and biological data, may provide more confident conclusions.

Phylogenetic analysis based on the primary sequence of proteins using the same ML algorithm RAxML-ng and alignment using different algorithms resulted in different tree topologies. Bootstrap values were also low, and these can be explained by a low level of sequence similarity, due to ancient divergence of the main *Duplodnaviria* groups and a high mutation rate. Sequence conservation level was higher for terminase than for MCP. However, due to the modular evolution of viruses, which is inherent to various viral groups [14,92], using just one protein cannot uncover the evolutionary history. The example of *Pseudomonas* phage MD8, examined in this study, as well as earlier [18], shows a different evolutionary history for MCP and terminase, as indicated by both the structural comparisons of AF models and sequence-based phylogenetic analysis.

The results of most structural comparisons and phylogenies based on amino acid sequences seem to correlate for viruses that are evolutionarily closely related, such as T4-like phages. Both all-against-all structural comparison and sequence-based phylogenies grouped T4- and ϕ29-like viruses in monophyletic groups, and these results appear to be biologically reasonable. Larger proteins of evolutionarily related representatives of three *Herpesvirales* families [93,94] were better clustered using algorithms employing structural alignments of the predicted models. This is an argument in favour of using structural analysis, which can be explained by good modelling accuracy and the superior sensitivity of structural comparisons in comparison with sequence-based phylogeny. The DALI can be more appropriate than mTM-align for such purposes. DALI, and mTM to a lesser degree, demonstrated consistency with sequence-based phylogenetic analysis for relatively small distances estimated with the tree’s branch length (e.g., T4-like viral families), and better clustered the distant representatives of the *Hepresvirales* families. Therefore, it seems reasonable to suggest that structural comparisons based on AF predictions can also work in the context of intermediate distances. It is probable that the detailed examination of protein evolution, (e.g., the emergence of new domains and subdomains inherent for the whole group), may assist evolutionary analysis. This approach was used in the analysis of the evolution of phage tail sheath protein [95], featuring the subsequent adding of new domains while maintaining the common conserved domain. Interestingly, the predicted structures of the modelled MCPs of *Crassvirales* phages and group 3 phages (*Pachyviridae* and *Pervagoviridae* representatives) contain similar additional domains, composed mainly of β-strands, which supports the suggestion of their relatedness.

It is extremely difficult to understand the evolutionary history of viral groups, and the corresponding evolutionary-based taxonomic updates, using only one protein, but a set of conserved marker genes with the same origin could indicate evolutionary relationships and taxonomic classification. Such an approach is widely used, and might include various groups of DNA viruses [96,97,98], but difficulties might be encountered with *Duplodnaviria* viruses, because of the limited number of shared genes and recombination events, as a result of modular evolution. For instance, many phages lack some replication protein genes [99], making it impossible to use them in phylogenetic analysis. The approach of using the main clustering tools, such as predicted proteome-based clustering tools [2], to reconstruct evolutionary history at the level of families could hypothetically lead to a situation where newly acquired genes can mask highly mutated conserved genes, leading to erroneous conclusions about the origin of these groups.

Viral evolution is characterised by network character [100,101], and genomics-based evaluations of evolutionary history, using gene network clustering, do show the connections between related groups; they do not, however, readily show the history of the appearance of these connections. It is probable that some reference points are needed for the assessment of *Duplodnaviria* evolution and, in genome-based approaches, they should include proteins of common origin for all viruses represented.

Recent meticulous work carried out on the classification of archaeal viruses [24] has laid the foundations for classification of this important viral group. The analysis of all available complete genomes of archaeal head-tailed viruses has made it possible to make assumptions about the ancient divergence of archaeal and bacterial tailed viruses and the intensive exchange of genes involved in DNA metabolism and counter-defence mechanisms. The results of the present study, including structural comparisons and sequence-based phylogenies, indicate that such assumptions may be needed in some modifications, such as those explaining the non-monophyletic character of the relationships of archaeal MCPs and terminases. The fact that a BLAST search indicated the closest related proteins not among representatives of the same order of head-tailed archaeal viruses, but among representatives of other taxa of archaeal and bacterial viruses, and the results of phylogenetic studies not in accordance with current classification, may indicate a more complex pattern of early evolution of *Caudoviricetes* viruses. Otherwise, the requirements of monophyly for orders [2] should be explained or clarified.

Apparently, the theory and practice of the evolutionary taxonomy of *Duplodnaviria* viruses needs further clarification and refinements, which should be based on decisions about the priorities relating to various genomic data, decisions that have several aspects, including philosophical questions about the relationship between holistic and reductionist approaches in evolutionary biology. The evolution of genome organisation, the emergence and exchange of genomic modules and the evolution of conserved proteins can, together, be used for the evolutionary taxonomy of viruses.

Given the lack of experimental data on viral proteins, accurate predictions of AlphaFold can be useful for reconstructing the evolution of proteins, making AlphaFold an important tool in evolutionary taxonomy. AlphaFold modelling can also be used to functionally assign proteins when commonly used BLAST and HMM searches fail. The collection of data obtained during this study, involving AlphaFold predictions and sequence-based analysis, has made it possible for several suggestions to be made concerning refinements to present taxonomic classification:

1. Bacteriophages of *Guelinviridae*, *Rountreeviridae* and *Salasmaviridae* families, *Curtobacterium* phage Ayka (group 1) and related phages can be considered as candidates for the delineation of a new order.
2. The families *Ackermannviridae*, *Kyanoviridae* and *Straboviridae* (group 2), and related phages, can be assigned to a new taxon of a higher rank.
3. The bacteriophages of *Pachyviridae* and *Pervagoviridae* families (group 3) are related to *Crassvirales* phages. These, and related phages, can be considered as candidates for the delineation of a new order.
4. The bacteriophages of the *Casjensviridae* family and *Lambdavirus lambda* (group 4) are evolutionarily related. The taxonomy of these, and related, groups requires additional research and refinements, taking into account the specifics of the evolution of temperate phages, which are highly susceptible to genetic exchanges.
5. The bacteriophages of the *Duneviridae* and *Helgolandviridae* families (group 5) are evolutionarily related and, together with related phages, can be considered as candidates for the delineation of a new order.
6. The evolutionary history and taxonomic classification of head-tailed archaeal viruses requires additional research and further clarification.

## 5. Conclusions

AlphaFold’s highly accurate predictions create new possibilities for studying the evolutionary history of viral proteins. In this study, use of the results of AlphaFold modelling, combined with the results of sequence-based analysis and other data, enabled the discovery of deep evolutionary relationships and suggestions for possible upgrades to taxonomic classifications of *Duplodnaviria* viruses.

## Figures and Tables

**Table 1:** List of *Duplodnaviria Heunggongvirae* representative viruses taken for the analyses and general genomic features. Eukaryotic viruses are coloured blue, bacterial viruses are coloured green, and archaeal viruses are coloured yellow.

Species	Original Name	ICTV Taxonomy	Genome Size, b.p.	GC Content, %	NCBI Accession
*Ranid herpesvirus 1*	*Lucke tumor herpesvirus-ranid herpesvirus 1*	*Herpesvirales; Alloherpesviridae*	220,859	54.6	DQ665917.1
*Human alphaherpesvirus 1*	*Human herpesvirus 1* strain 17	*Herpesvirales; Herpesviridae*	152,222	68.3	JN555585.1
*Haliotid herpesvirus 1*	*Abalone herpesvirus* Victoria/AUS/2009	*Herpesvirales; Malacoherpesviridae*	211,518	46.8	JX453331.1
*Curtobacterium* phage *Ayka*	*Curtobacterium* phage Ayka	*not classified*	18,400	52.6	ON381767.1
*LacPavin*	*LacPavin*_0818_WC45	*not classified*	735,411	32.2	LR756501.1
*Pseudomonas* phage *MD8*	*Pseudomonas* phage MD8	*not classified*	43,277	61.1	KX198612.1
*Limestonevirus limestone*	*Dickeya* phage vB-DsoM-LIMEstone1	*Ackermannviridae*	152,427	49.3	HE600015.1
*Harrekavirus harreka*	*Olleya* phage Harreka_1	*Aggregaviridae*	43,175	32.0	MT732457.1
*Cebadecemvirus phi10una*	*Cellulophaga* phage phi10:1	*Assiduviridae*	53,664	31.5	KC821618.1
*Teseptimavirus T7*	*Escherichia* phage T7	*Autographiviridae*	39,937	48.4	V01146.1
*Chivirus chi*	*Salmonella* phage χ	*Casjensviridae*	59,407	56.5	JX094499.1
*Lambdavirus lambda*	*Escherichia* phage λ	*Lambdavirus*	48,502	49.9	J02459.1
*Suwonvirus PP101*	*Pectobacterium* phage PP101	*Chaseviridae*	53,333	44.9	KY087898.2
*Junduvirus communis*	uncultured phage cr2_1	*Crassvirales; Crevaviridae*	95,815	32.7	MZ130489.1
*Jahgtovirus gastrointestinalis*	uncultured phage cr36_1	*Crassvirales; Intestiviridae*	96,466	32.0	MZ130479.1
*Kahnovirus copri*	uncultured phage cr44_1	*Crassvirales; Steigviridae*	93,564	35.8	MZ130483.1
*Afonbuvirus coli*	uncultured phage cr35_1	*Crassvirales; Suoliviridae*	97,706	31.4	MZ130499.1
*Cetovirus ceto*	*Vibrio* phage Ceto	*Demerecviridae*	128,241	39.9	MG649966.1
*Donellivirus gee*	*Bacillus* phage G	*Donellivirus*	497,513	29.9	JN638751.1
*Tunavirus T1*	*Escherichia* phage T1	*Drexlerviridae*	48,836	45.6	AY216660.1
*Unahavirus uv1H*	*Flavobacterium* phage 1H	*Duneviridae*	39,290	31.4	KU599889.1
*Freyavirus freya*	*Polaribacter* phage Freya_1	*Forsetiviridae*	43,978	28.9	MT732463.1
*Gregsiragusavirus CPS1*	*Clostridium* phage CPS1	*Guelinviridae*	19,089	28.3	KY996523.1
*Leefvirus Leef*	*Polaribacter* phage Leef_1	*Helgolandviridae*	37,547	29.7	MT732473.1
*Byrnievirus HK97*	*Escherichia* phage HK97	*Hendrixvirinae*	39,732	49.8	AF069529.1
*Pecentumvirus P100*	*Listeria* phage P100	*Herelleviridae*	131,384	36.0	DQ004855.1
*Beejeyvirus BJ1*	*Halorubrum* virus BJ1	*Kirjokansivirales; Graaviviridae*	42,271	64.9	AM419438.1
*Retbasiphovirus HFTV1*	*Haloferax* tailed virus 1	*Kirjokansivirales; Haloferuviridae*	38,059	54.1	MG550112.1
*Hatrivirus HATV3*	*Haloarcula* tailed virus 3	*Kirjokansivirales; Pyrstoviridae*	42,293	51.1	MZ334527.1
*Lonfivirus HSTV1*	*Haloarcula sinaiiensis* tailed virus 1	*Kirjokansivirales; Shortaselviridae*	32,189	60.3	KC117378.1
*Bellamyvirus bellamy*	*Synechococcus* phage Bellamy	*Kyanoviridae*	204,930	41.1	MF351863.1
*Clampvirus HHTV1*	*Haloarcula hispanica* tailed virus 1	*Madisaviridae*	49,107	56.5	KC292025.1
*Pseudomonas virus Yua*	*Pseudomonas* phage YuA	*Mesyanzhinovviridae*	58,663	64.3	AM749441.1
*Metforvirus Drs3*	*Methanobacterium* virus Drs3	*Methanobavirales; Anaerodiviridae*	37,129	41.2	MH674343.1
*Psimunavirus psiM2*	*Methanobacterium* phage psiM2	*Methanobavirales; Leisingerviridae*	26,111	46.3	AF065411.1
*Mollyvirus colly*	*Maribacter* phage Colly_1	*Molycolviridae*	124,169	36.3	MT732450.1
*Noahvirus arc*	Bacteriophage DSS3_VP1	*Naomviridae*	75,087	47.5	MN602266.1
*Bonaevitae bonaevitae*	*Microbacterium* phage BonaeVitae	*Orlajensenviridae*	17,451	68.2	MH045556.1
*Bacelvirus phi46tres*	*Cellulophaga* phage phi46:3	*Pachyviridae*	72,961	32.7	KC821622.1
*Peduovirus P2*	Bacteriophage P2	*Peduoviridae; Peduovirus*	33,593	50.2	AF063097.1
*Callevirus Calle*	*Cellulophaga* phage Calle_1	*Pervagoviridae*	72,979	38.1	MT732432.1
*Phikzvirus phiKZ*	*Pseudomonas* phage phiKZ	*Phikzvirus*	280,334	36.8	AF399011.1
*Rosenblumvirus rv66*	Bacteriophage 66	*Rountreeviridae*	18,199	29.3	AY954949.1
*Salasvirus phi29*	*Bacillus* phage phi29	*Salasmaviridae*	19,282	40.0	EU771092.1
*Halohivirus HHTV2*	*Haloarcula hispanica* tailed virus 2	*Saparoviridae*	52,643	66.6	KC292024.1
*Enquatrovirus N4*	*Escherichia* phage N4	*Schitoviridae*	70,153	41.3	EF056009.1
*Tequatrovirus T4*	*Escherichia* phage T4	*Straboviridae*	168,903	35.3	AF158101.6
*Pormufvirus HRTV28*	*Halorubrum* tailed virus 28 isolate HRTV-28/28	*Suolaviridae*	35,270	64.3	MZ334528.1
*Hacavirus HCTV1*	*Haloarcula californiae* tailed virus 1	*Thumleimavirales; Druskaviridae*	103,257	57.0	KC292029.1
*Haloferacalesvirus HF1*	*Halophage* HF1	*Thumleimavirales; Hafunaviridae*	75,898	55.8	AY190604.2
*Hagravirus HGTV1*	*Halogranum* tailed virus 1	*Thumleimavirales; Halomagnusviridae*	143,855	50.4	KC292026.1
*Eilatmyovirus HATV2*	*Haloarcula* tailed virus 2	*Thumleimavirales; Soleiviridae*	63,301	49.7	MZ334525.1
*Myohalovirus phiH*	*Halobacterium* phage phiH	*Vertoviridae*	58,072	63.7	MK002701.1
*Bromdenvirus bromden*	*Mycobacterium* phage Bromden	*Vilmaviridae*	70,183	58.2	MH576973.1
*Peternellavirus peternella*	*Winogradskyella* phage Peternella_1	*Winoviridae*	39,649	35.4	MT732475.1
*Foxborovirus foxboro*	*Gordonia* phage Foxboro	*Zierdtviridae*	67,773	65.8	MH727547.1
*Siovirus americense*	*Roseobacter* phage SIO1	*Zobellviridae*	39,898	46.2	AF189021.1

## Data Availability

Not applicable.

## Supplementary Materials

The following supporting information can be downloaded at: https://www.mdpi.com/article/10.3390/biom13010110/s1. Figure S1: VIRIDIC-generated heatmap of 57 viruses representing different *Duplodnaviria* groups. The colour coding indicates the clustering of phage genomes based on intergenomic similarity. The numbers represent the similarity values for each genome pair, rounded to the first decimal. Figure S2: Structural models of the product of whole genes encoding for the major capsid proteins in genomes of representative viruses (Table 1), obtained with AlphaFold. The N-terminus is labelled with a blue circle and the C-terminus is labelled with a yellow circle. Figure S3: Structural models of the product of whole genes encoding for the ATPase subunit of terminase in genomes of representative viruses (Table 1), obtained with AlphaFold. The N-terminus is labelled with a blue circle and the C-terminus is labelled with a yellow circle. Figure S4: The DALI matrix based on pairwise Z-score comparisons of 57 AF models of major capsid protein, with removed parts supposedly belonging to the protease and scaffolding domains and encapsulin. Figure S5: Best-scoring ML phylogenetic tree constructed with 57 amino acid sequences of major capsid protein and an encapsulin aligned with MAFFT. The scale bar shows 0.5 estimated substitutions per site and the trees were rooted to encapsulin. Figure S6: Best-scoring ML phylogenetic tree constructed with 57 amino acid sequences of major capsid protein and an encapsulin aligned with MUSCLE. The scale bar shows 0.5 estimated substitutions per site and the trees were rooted to encapsulin. Figure S7: Best-scoring ML phylogenetic tree constructed with 57 amino acid sequences of major capsid protein and an encapsulin aligned with mTM-align. The scale bar shows 0.5 estimated substitutions per site and the trees were rooted to encapsulin. Figure S8: Best-scoring ML phylogenetic tree constructed with 57 amino acid sequences of ATPase subunits of *Heunggongvirae* terminases and an *Adenoviridae* terminase aligned with MAFFT. The numbers near the tree branches indicate the TBE support. The total number of bootstrap trees was 1000. The scale bar shows 0.5 estimated substitutions per site and the trees were rooted to *Adenoviridae*. Figure S9: Best-scoring ML phylogenetic tree constructed with 57 amino acid sequences of ATPase subunits of *Heunggongvirae* terminases and an *Adenoviridae* terminase aligned with MUSCLE. The numbers near the tree branches indicate the TBE support. The total number of bootstrap trees was 1000. The scale bar shows 0.5 estimated substitutions per site and the trees were rooted to *Adenoviridae*.; Figure S10: Best-scoring ML phylogenetic tree constructed with 57 amino acid sequences of ATPase subunits of *Heunggongvirae* terminases and an *Adenoviridae* terminase aligned with mTM-align. The numbers near the tree branches indicate the TBE support. The total number of bootstrap trees was 1000. The scale bar shows 0.5 estimated substitutions per site and the trees were rooted to *Adenoviridae*. Figure S11: GRAViTy dendrogram constructed with database “DB-B: Baltimore Group Ib-Prokaryotic and archaeal dsDNA viruses (VMRv34)” and genomic sequences of 57 representative viruses.
