# Supplementary figures and images for: Use of an Integrated Approach Involving AlphaFold Predictions for the Evolutionary Taxonomy of Duplodnaviria Viruses
Source: Biomolecules. 2023 Jan 5;13(1):110. doi: 10.3390/biom13010110 (PMC9855967; doi:10.3390/biom13010110)

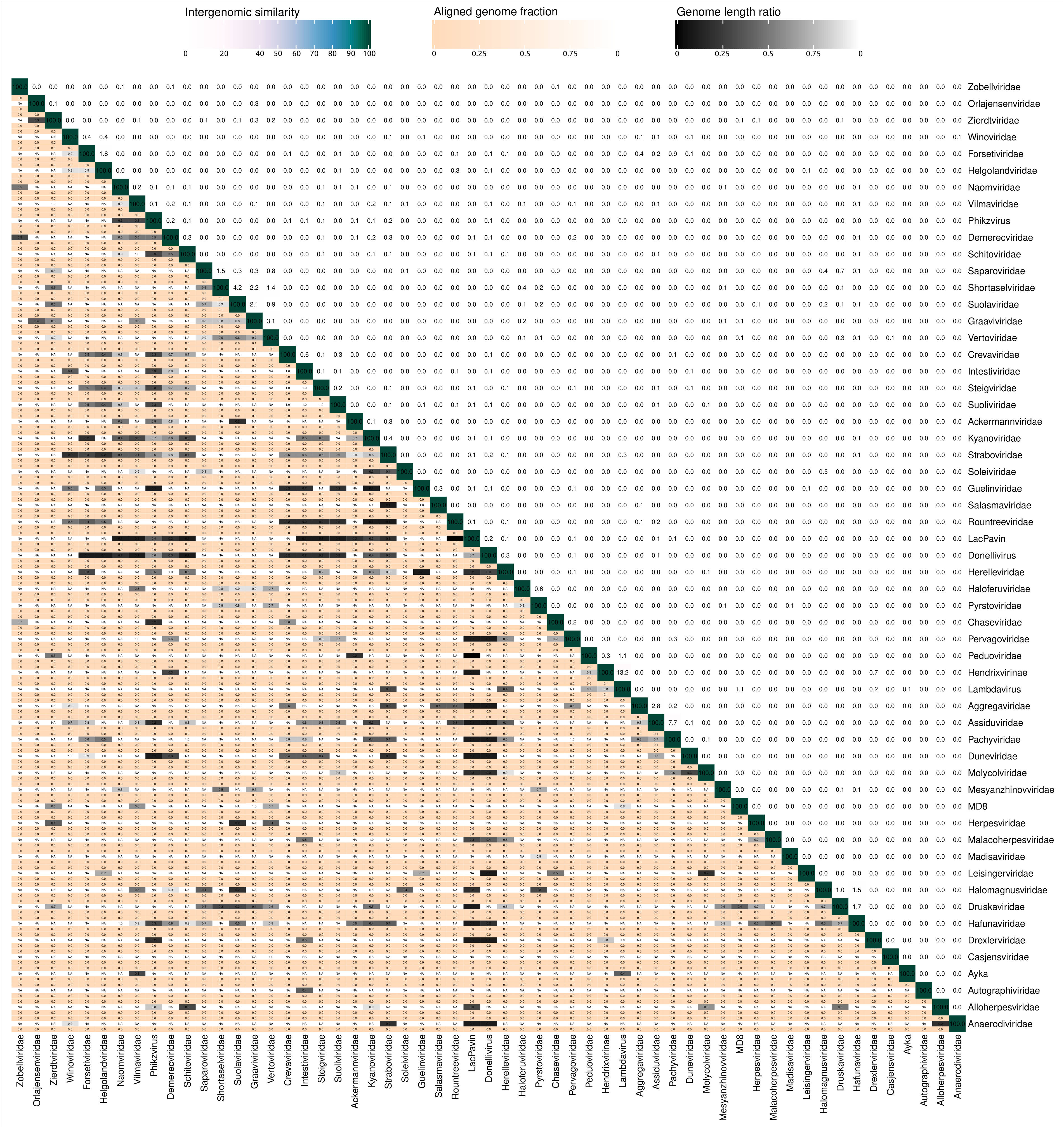

Supplement: Supplementary file 1 [file biomolecules-13-00110-s001.zip › Figure_S1.jpg]

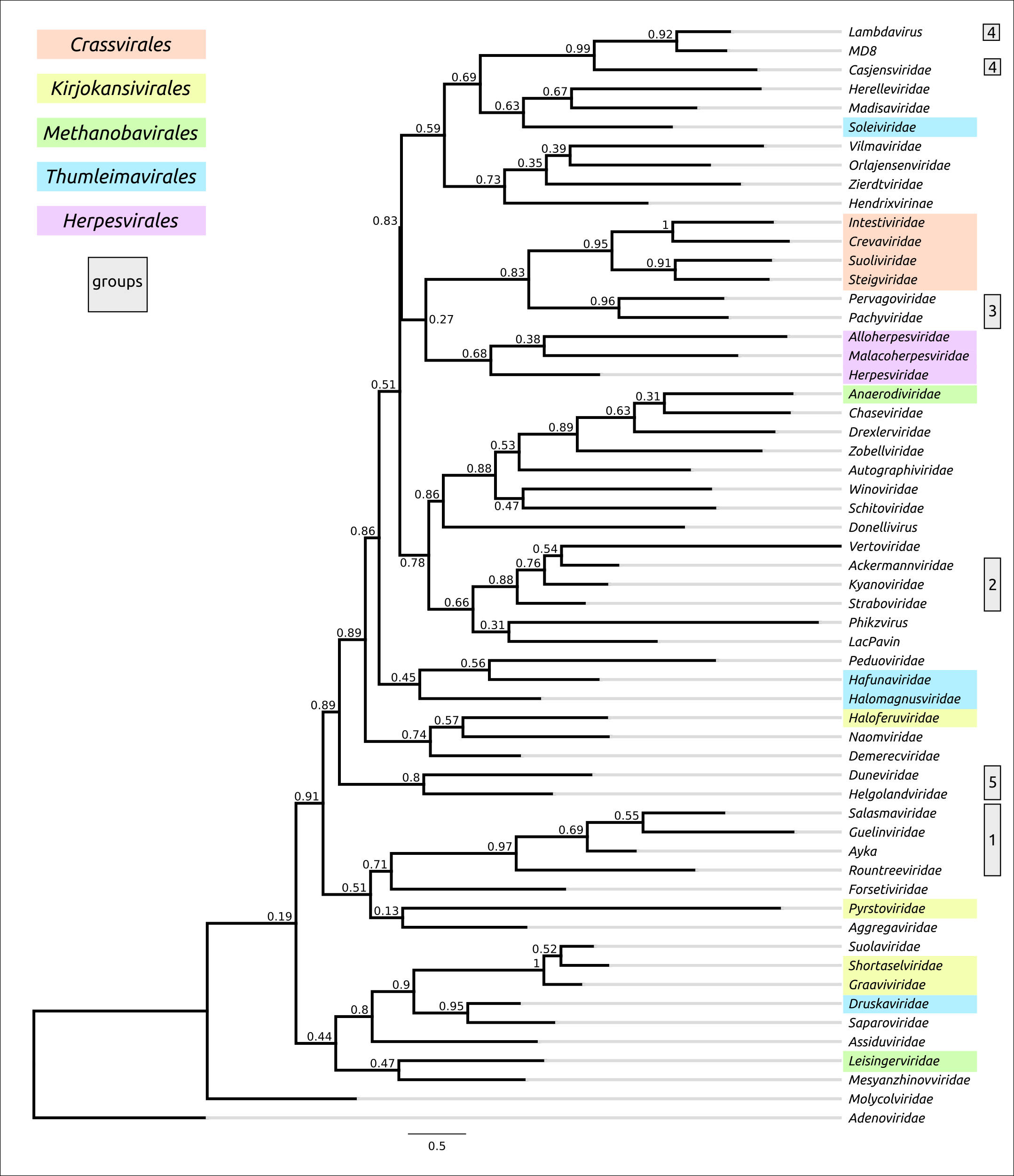

Supplement: Supplementary file 1 [file biomolecules-13-00110-s001.zip › Figure_S10.jpg]

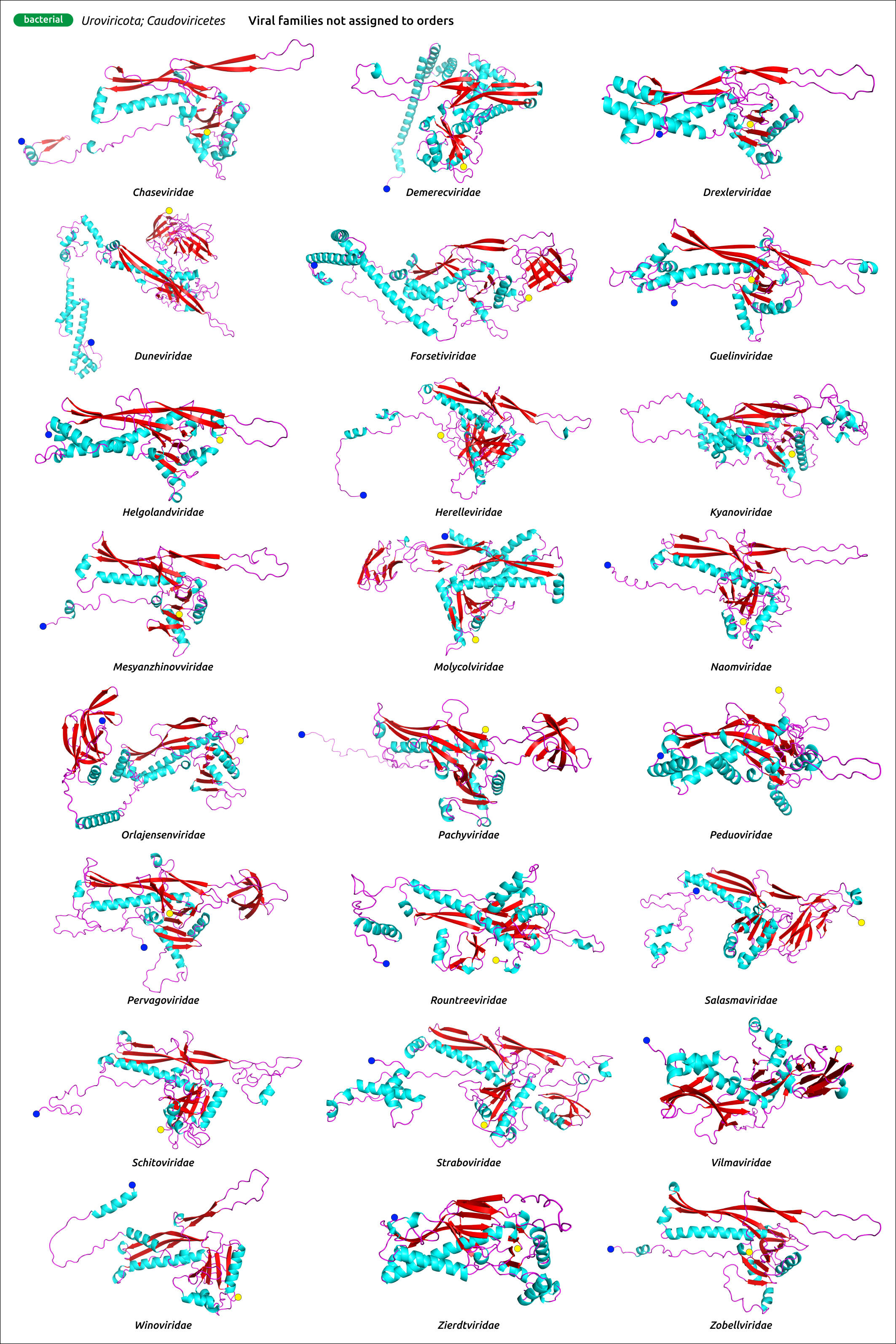

Supplement: Supplementary file 1 [file biomolecules-13-00110-s001.zip › Figure_S2-1.jpg]

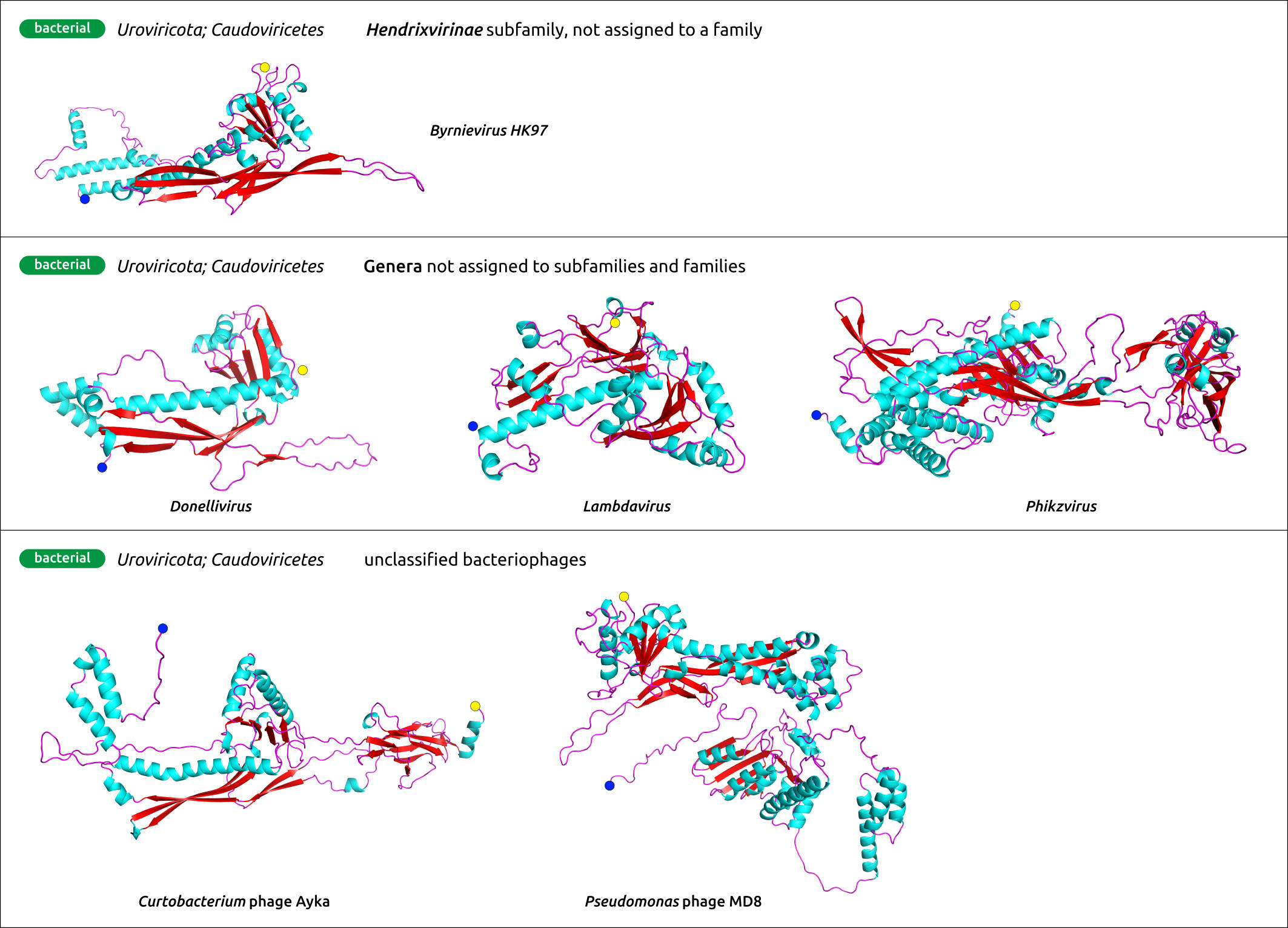

Supplement: Supplementary file 1 [file biomolecules-13-00110-s001.zip › Figure_S2-2.jpg]

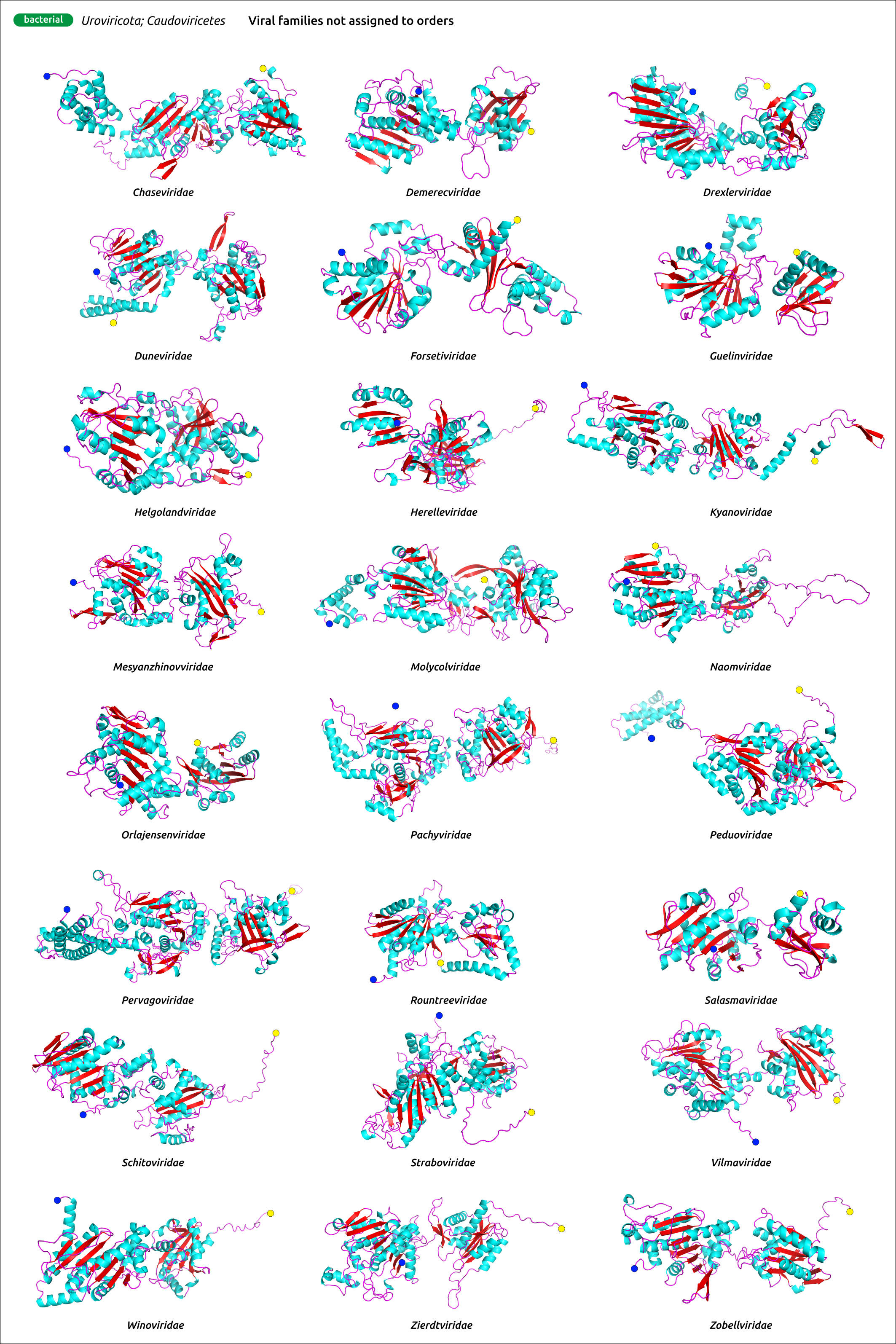

Supplement: Supplementary file 1 [file biomolecules-13-00110-s001.zip › Figure_S3-1.jpg]

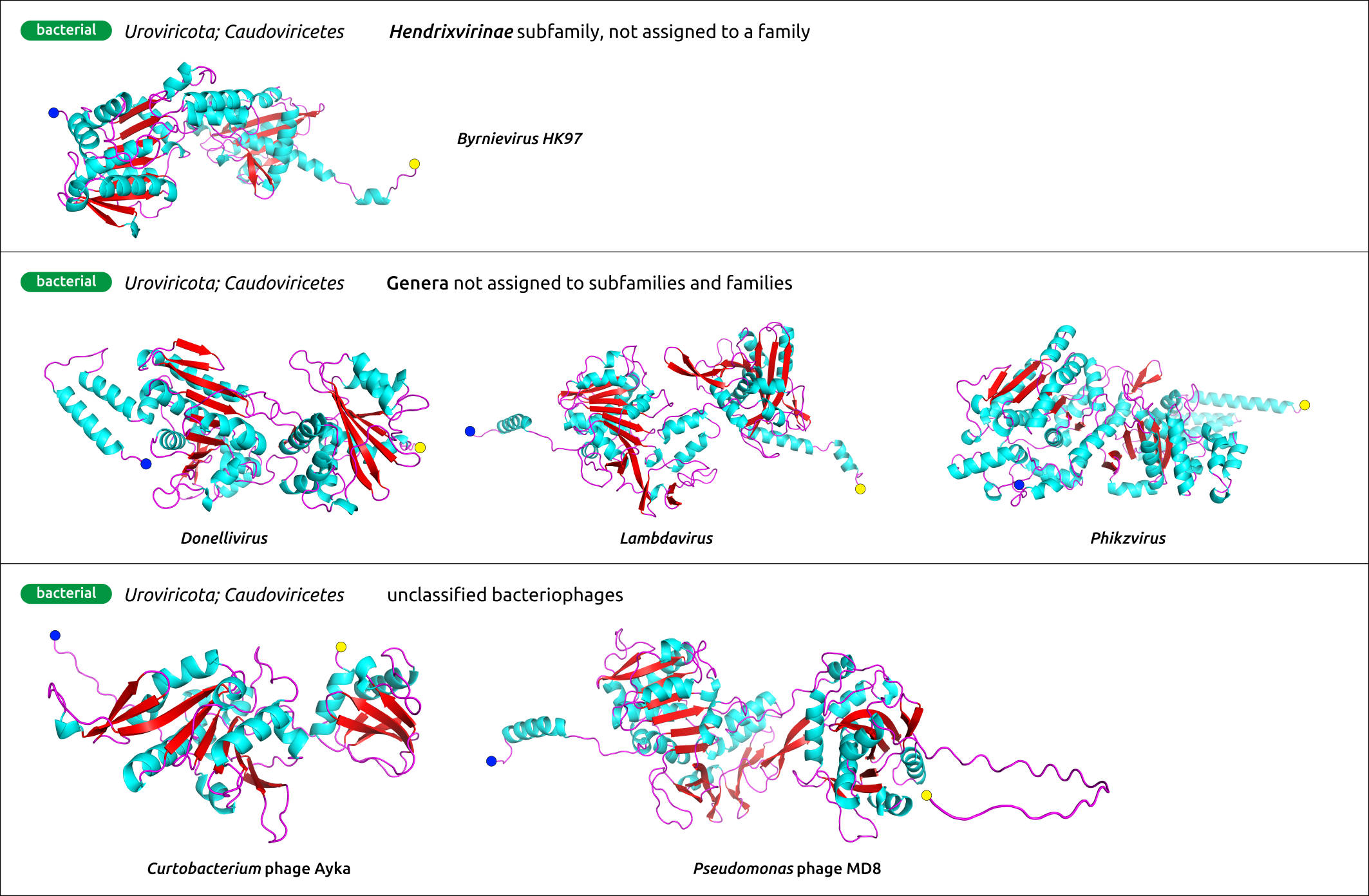

Supplement: Supplementary file 1 [file biomolecules-13-00110-s001.zip › Figure_S3-2.jpg]

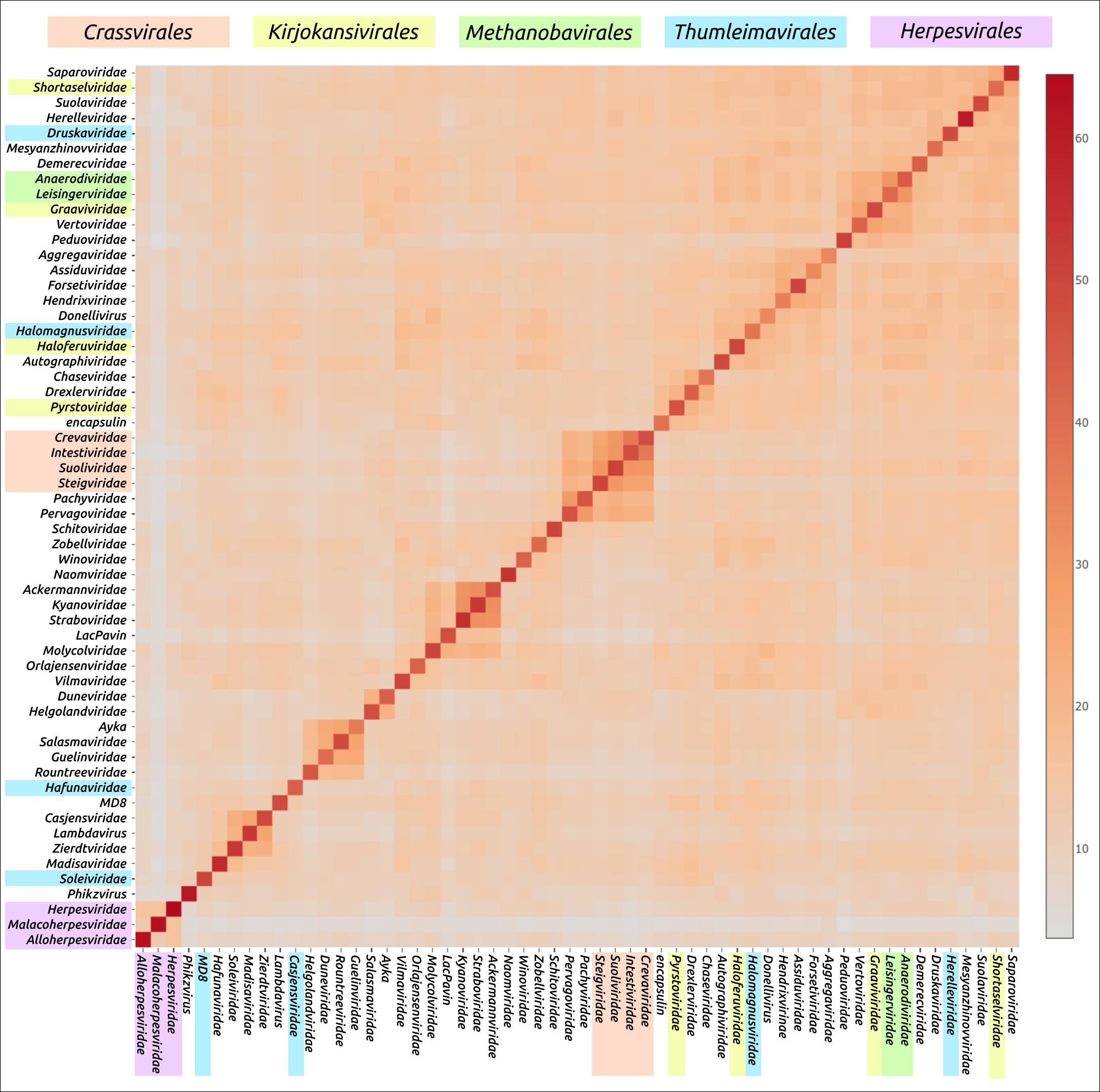

Supplement: Supplementary file 1 [file biomolecules-13-00110-s001.zip › Figure_S4.jpg]

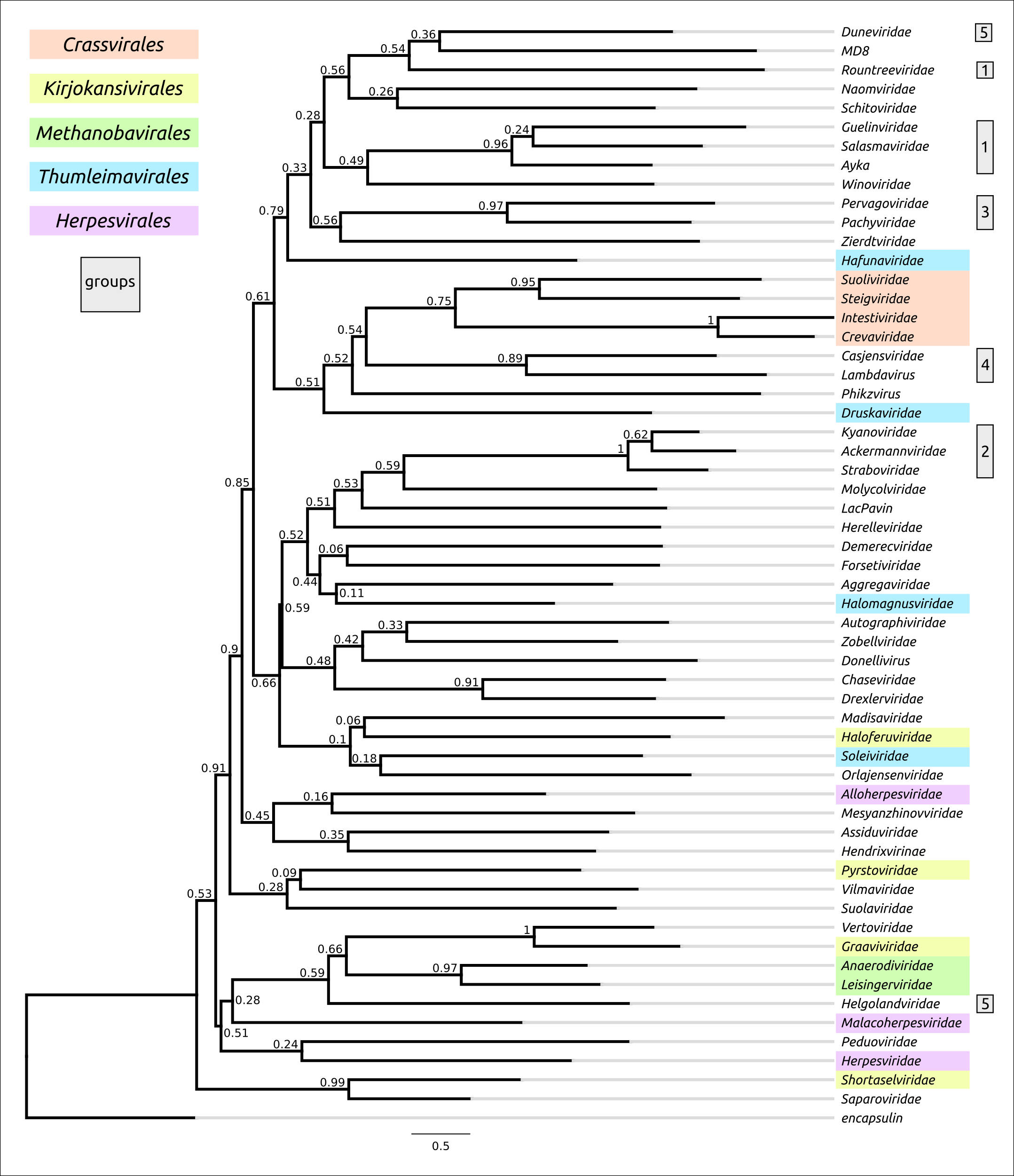

Supplement: Supplementary file 1 [file biomolecules-13-00110-s001.zip › Figure_S5.jpg]

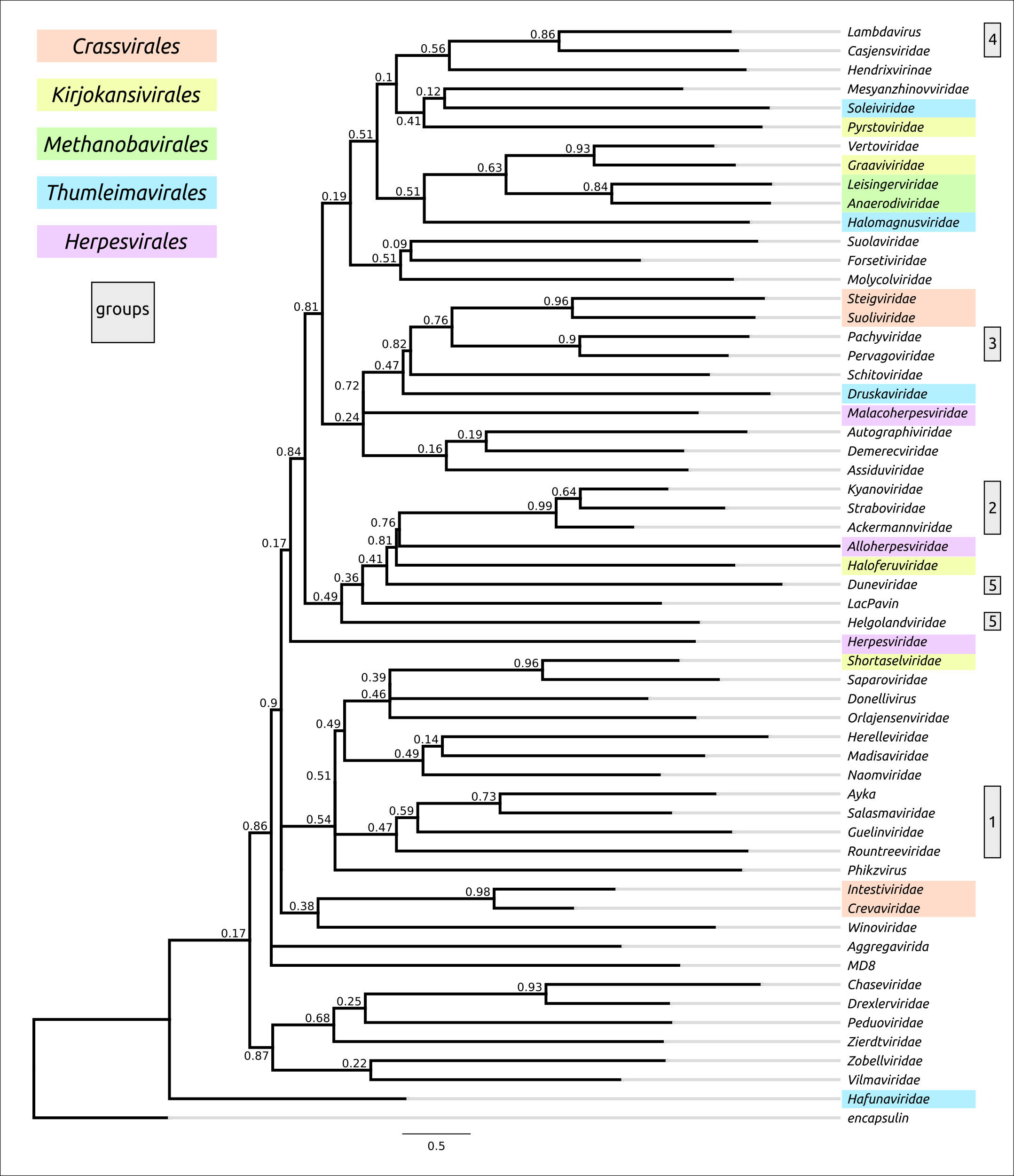

Supplement: Supplementary file 1 [file biomolecules-13-00110-s001.zip › Figure_S6.jpg]

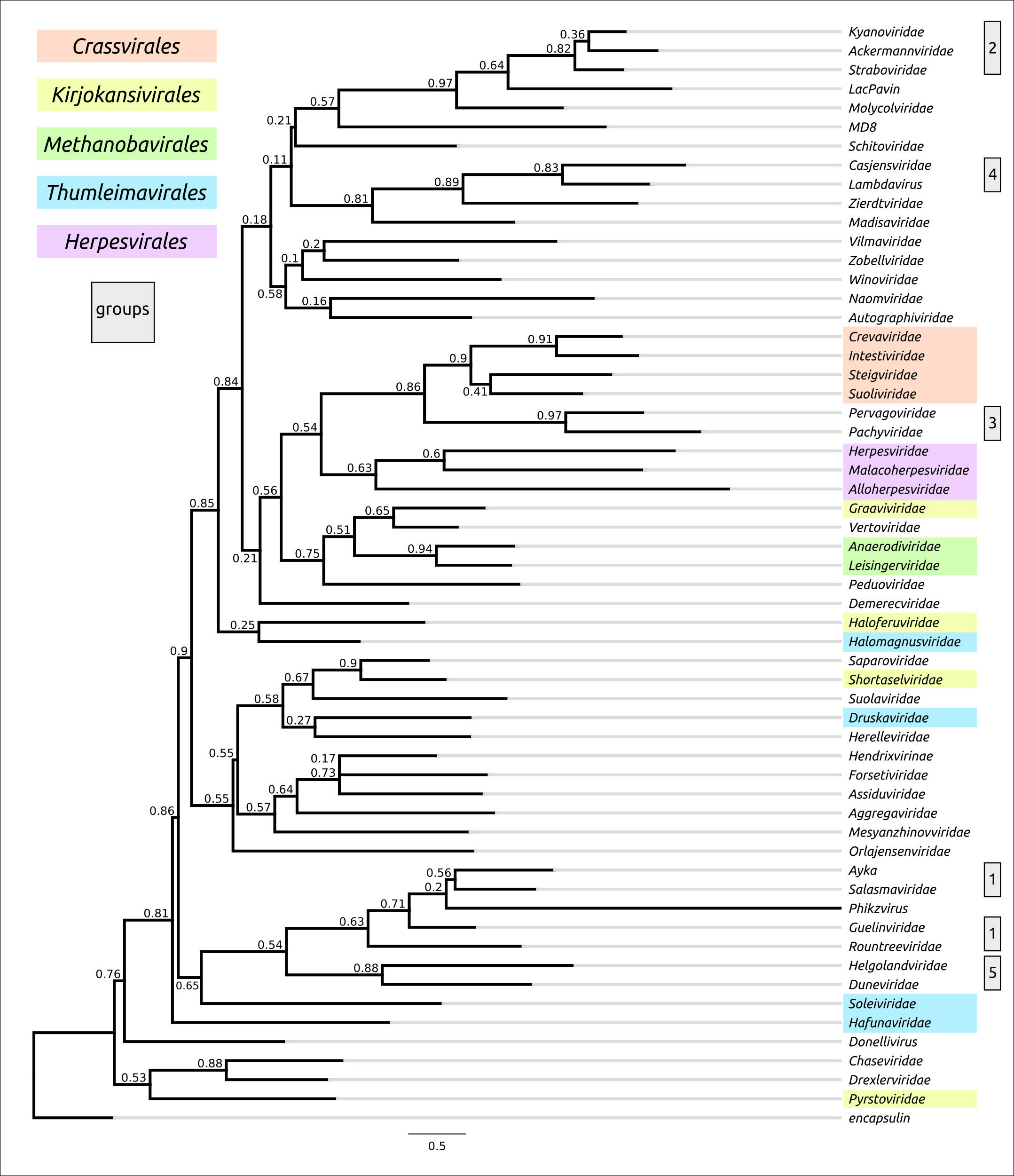

Supplement: Supplementary file 1 [file biomolecules-13-00110-s001.zip › Figure_S7.jpg]

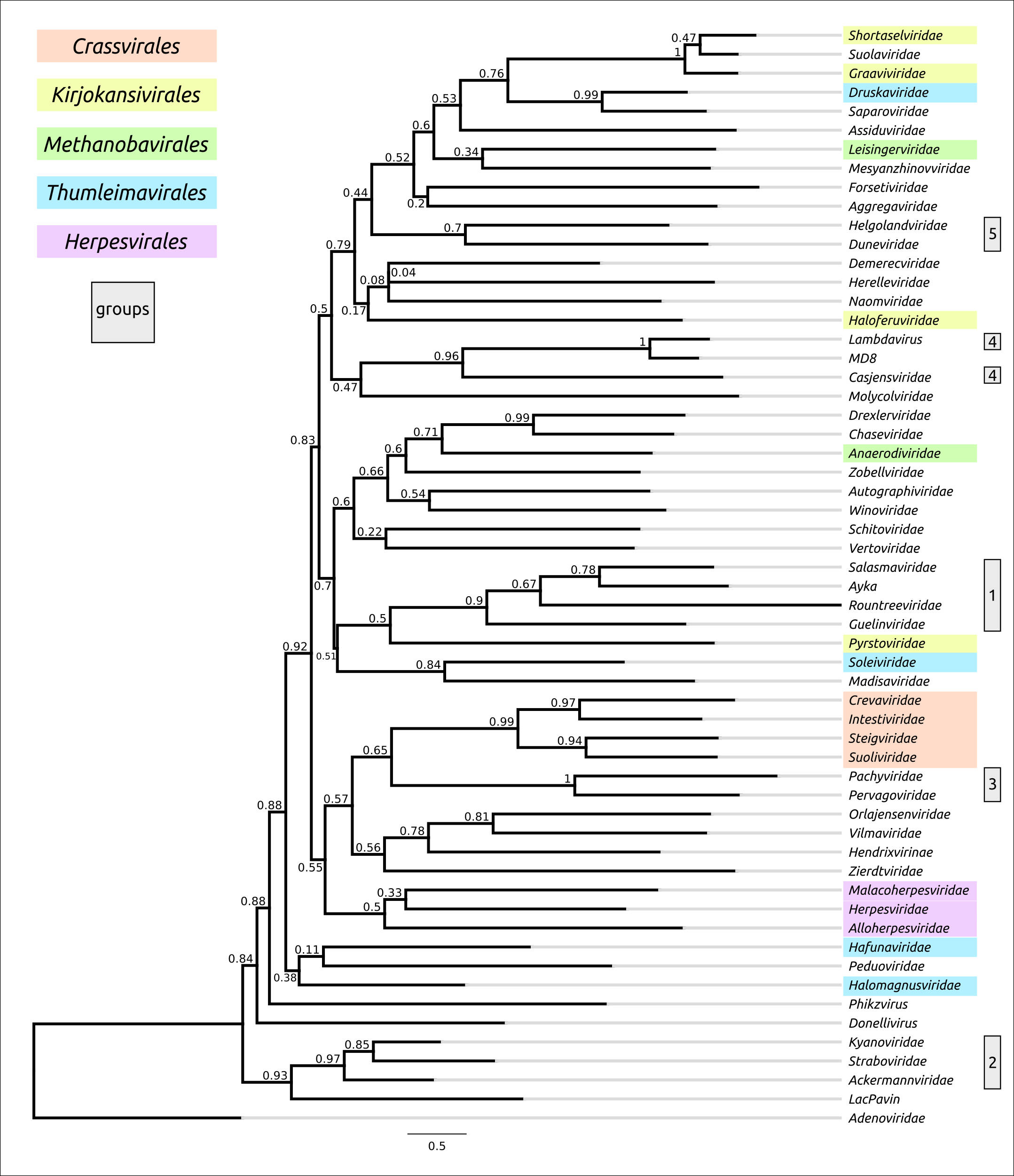

Supplement: Supplementary file 1 [file biomolecules-13-00110-s001.zip › Figure_S8.jpg]

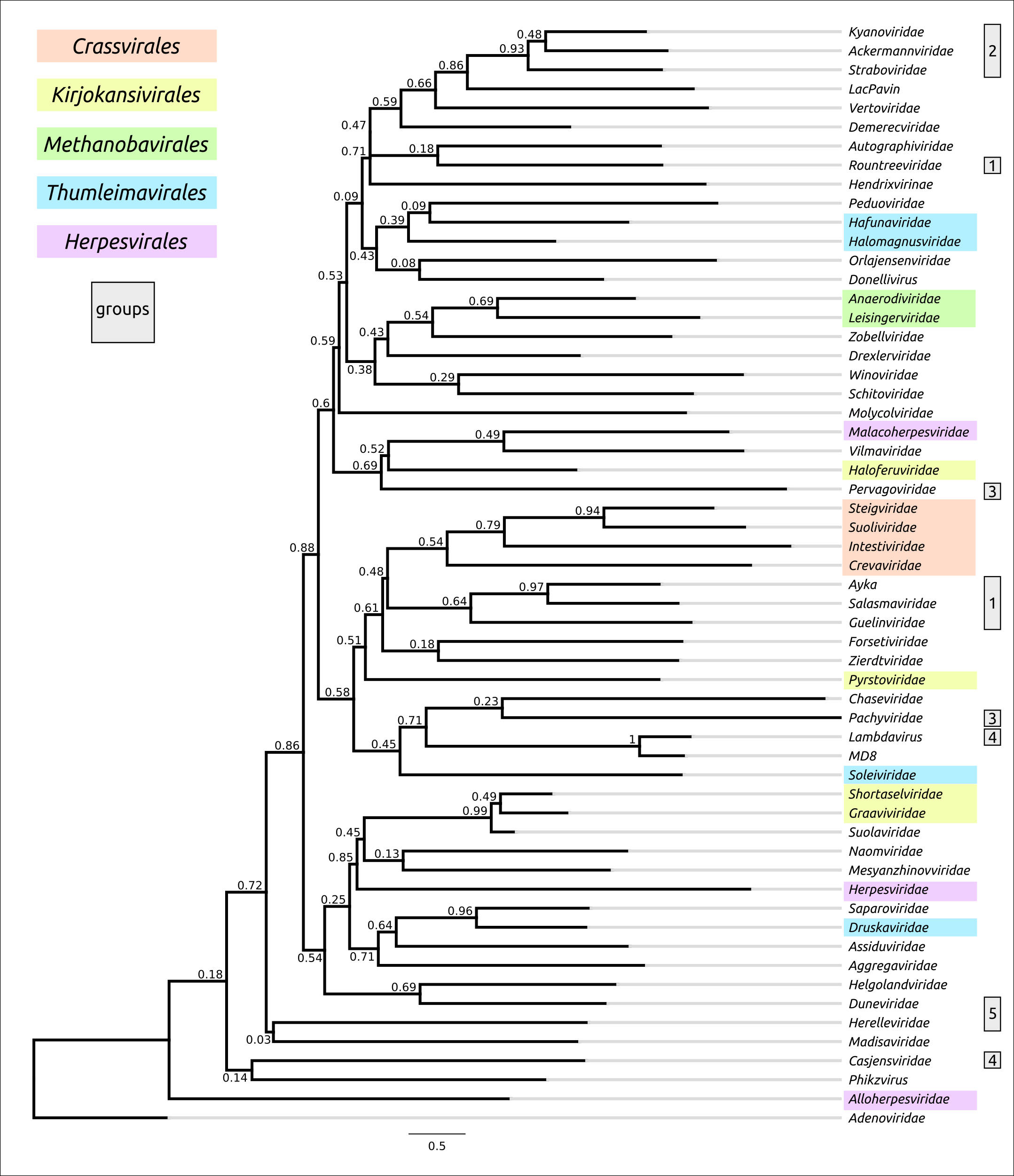

Supplement: Supplementary file 1 [file biomolecules-13-00110-s001.zip › Figure_S9.jpg]
